# RNA Sequencing and Co-expressed Long Non-coding RNA in Modern and Wild Wheats

**DOI:** 10.1038/s41598-017-11170-8

**Published:** 2017-09-06

**Authors:** Halise Busra Cagirici, Burcu Alptekin, Hikmet Budak

**Affiliations:** 1 Sabanci University, Molecular Biology, Genetics and Bioengineering Program, Istanbul, Turkey; 2 Cereal Genomics Lab, Montana State University, Department of Plant Sciences and Plant Pathology, Bozeman, MT USA

## Abstract

There is an urgent need for the improvement of drought-tolerant bread and durum wheat. The huge and complex genome of bread wheat (BBAADD genome) stands as a vital obstruction for understanding the molecular mechanism underlying drought tolerance. However, tetraploid wheat (*Triticum turgidum ssp*., BBAA genome) is an ancestor of modern bread wheat and offers an important model for studying the drought response due to its less complex genome. Additionally, several wild relatives of tetraploid wheat have already shown a significant drought tolerance. We sequenced root transcriptome of three tetraploid wheat varieties with varying stress tolerance profiles, and built differential expression library of their transcripts under control and drought conditions. More than 5,000 differentially expressed transcripts were identified from each genotype. Functional characterization of transcripts specific to drought-tolerant genotype, revealed their association with osmolytes production and secondary metabolite pathways. Comparative analysis of differentially expressed genes and their non-coding RNA partners, long noncoding RNAs and microRNAs, provided valuable insight to gene expression regulation in response to drought stress. LncRNAs as well as coding transcripts share similar structural features in different tetraploid species; yet, lncRNAs slightly differ from coding transcripts. Several miRNA-lncRNA target pairs were detected as differentially expressed in drought stress. Overall, this study suggested an important pool of transcripts where their manipulations confer a better performance of wheat varieties under drought stress.

## Introduction

Wheat (*Triticum ssp*.) is one of the major sources of continuously increasing food demand, ranking third in crop production worldwide^1^. Domestication and cultivation efforts of agricultural practices resulted in an increased yield^2^ with the approximate global production of 700 million tons per year distributed over 200 million hectares^1^. Despite this spread distribution, obtained rate of yield is not sufficient to meet world food demand since production rate is significantly limited by biotic and abiotic stress factors^3^. Drought is one of the major abiotic stress factors worldwide, causing decrease in grain quality and yield loss through all cereals, including wheat^4^. Recent studies have suggested a substantial increase in drought caused by climate change and global warming^5^. In order to maintain sufficient amount of yield with an improved nutritional quality, development of new wheat varieties with an increased drought tolerance is urgently needed toward future challenges.

Wild plants evolved sophisticated stress tolerance and adaptation mechanisms to drought where the domestication of modern wheat varieties has led to the loss of these valuable genes in the process of domestication^6^. Introgression of the valuable elements from wild relatives has been an attractive approach for agronomical improvement of modern wheat varieties for decades^7^, due to their rich gene pool for the resistance to many different stress factors. Tetraploid emmer wheat (*T. turdigum ssp. dicoccoides*, 2n = 28, AABB) is the wild progenitor of the allohexaploid bread wheat (*T. aestivum*, 6n = 42, AABBDD) and the domesticated tetraploid durum wheat (*T. turgidum ssp. durum*, 4n = 24, AABB)^2^. Recent studies on tetraploid wheat varieties revealed contrasting drought tolerance in tetraploid wild emmer wheat varieties and domesticated tetraploid durum wheat^8, 9^. Ergen and colleagues surveyed drought response of several genotypes of wild and domesticated tetraploid wheat varieties; they were able to show that wild emmer wheat, genotype TR39477, exhibits the highest drought tolerance while TTD-22 genotype has the lowest tolerance under drought stress. On the other hand, durum wheat variety Kiziltan showed a moderate tolerance in response to slow drought imposition^9^; however, complete mechanism of these drought responses remains elusive. A better understanding of the genomic background and the molecular mechanisms of drought responses in wild progenitors of wheat might reveal such favorable regulatory elements lost during domestication and cultivation processes.

In recent years, technological advances have made road into the reduction in the cost of sequencing experiments and availability of several genomic and transcriptomic data from bread wheat and its relatives/progenitors^10, 11^. Particularly, transcriptomic studies shed light into the differential expression and regulation of several transcripts under biotic and abiotic stress conditions, which further provide insights about the molecular mechanism associated with stress tolerance^4, 12, 13^. Total transcriptome sequencing and annotation possess a potential for detection of protein coding transcripts, differentially regulated under stress conditions, together with their non-coding interacting partners which is associated with a large portion of transcriptomes^14^. The content and the amount of the non-coding RNAs (ncRNAs) in the genome show an increased correspondence with the genome complexity which further supports their regulatory roles^15, 16^. Over the last decades, extensive studies in both animals and plants have shed light into the functions and mechanisms of ncRNAs such as microRNAs (miRNAs) and small interfering RNAs (siRNAs) in the transcriptional and post-transcriptional regulation of gene expression^17, 18^. While the miRNAs and siRNAs are referred as small RNAs (sRNAs) based on their small length ranging between 18 to 24 nucleotides, another type of ncRNAs longer than 200 nucleotides has been recently defined as long non-coding RNAs (lncRNAs)^19, 20^. LncRNAs resemble messenger RNAs (mRNAs) in their structure and biogenesis process, i.e., they are mainly transcribed by RNA Pol II and poly-adenylated, as though mRNAs^20^. Additionally, they might possess multiple exons and are subjected to alternative splicing. The major factor distinguishing lncRNAs from mRNAs is the lack of discernable coding potential of lncRNAs^21^. Besides, lncRNAs are composed of ~3 exons on average as opposed to ~11 exons in mRNAs and exhibit a more tissue-specific expression pattern compared to mRNAs where their expression is also relatively less than mRNAs in a given tissue^21^.

Emerging evidence has suggested that lncRNAs have regulatory roles in the major biological processes such as development, vernalization, nodulation and environmental stress adaptation both in direct and indirect manner^20^. As an example, two lncRNAs, the long antisense intragenic RNA (COOLAIR) and the intronic noncoding RNA (COLDAIR), have been detected as mediating the flowering process in *Arabidopsis* through silencing and epigenetic repression of Flowering Locus C (FLC)^22^. Additionally, several studies evinced the functions of lncRNAs in the biogenesis and targeting process of small-noncoding RNAs by possessing miRNA-siRNA precursor potential and sRNA target mimicry^19^. RNA-dependent DNA Methylation (RdDM) in plants, for example, utilizes lncRNAs acting as precursors of siRNAs which later target lncRNAs acting as scaffold RNAs recruiting siRNA-AGO4 complex together with RDM1 (RNA-directed DNA Methylation 1) to a target genomic loci for DNA methylation-mediated silencing^23^. In another example, lncRNA IPS1 has been shown to inhibit miR399-mediated cleavage of PHO2 as a competitor for PHO2 mRNA^24^. LncRNAs have also been identified as differentially expressed under several stress conditions and their regulation on both mRNA and sRNA pool detected as critical for stress tolerance and maintenance of vitality^25^; however, not that much effort has been done in drought responsive lncRNAs and their association with coding and other non-coding RNA species, particularly in cereals. Here, we present a detailed analysis of drought responsive mRNAs and lncRNAs along with their particular interaction with each other in three different tetraploid wheat varieties. Results revealed the presence of more than 200 putative stress responsive lncRNAs per cultivar which provided insights about drought tolerance mechanism in ancestor of modern wheat. Additionally, this study presents a brief method for precise identification and detailed characterization of lncRNAs for plants lacking both an annotated genome and a reference genome.

## Results

### *De novo* assembly of transcriptomes

In our previous study, two wild emmer wheat genotypes, *T. turgidum ssp. dicoccoides* TR39477 and TTD-22 showed marked differences in tolerance to drought stress when compared to the modern durum wheat, *T. turgidum ssp. durum var*. Kiziltan. Upon slow drought treatment, Kiziltan exhibited a moderate reaction whereas TR39477 and TTD-22 exhibited the most and the least tolerance, respectively^9^. High-throughput sequencing of root samples from control and drought-stressed Kiziltan, TR39477 and TTD-22 led to more than 27,000,000 raw sequence reads^8^. In order to remove adaptor sequences and perform the quality trimming, Trimmomatic^26^ analysis evaluated and more than 95% of raw reads were cleaned after initial processing with Trimmomatic (Table 1). The clean reads were assembled using Trinity software^27^ yielding a total of 243,670, 211,709 and 203,230 transcripts for Kiziltan, TR39477 and TTD-22, respectively. The average contig lengths detected as altering between 666 and 779 nucleotides long where the total transcriptome size ranges between 99.7 to 146.6 Mb (Table 1).

**Table 1:** Statistics on quality trimming and assembly construction for the transcriptome assemblies.

Samples	Kiziltan Control	Kiziltan Drought	TR39477 Control	TR39477 Drought	TTD-22 Control	TTD-22 Drought
*The quality trimming of samples*						
Before trimming	35,463,556	36,944,980	35,212,424	30,670,932	27,505,294	32,690,630
After trimming	33,772,655	35,171,816	33,698,813	29,223,580	26,200,743	31,249,427
*Assembly statistics for the samples*						
Number of transcripts	204128	18817	169762	159940	168314	155170
Median contig length (b)	482	516	478	483	468	494
Average contig	666.67	779.58	731.65	741.67	726.12	752.61
Total length (Mb)	99.78	146.69	129.32	125.24	122.22	116.78
GC%	49.72	49.45	49.98	49.92	50.63	50.25
N50	1024	1082	1001	1108	1004	1056

### Functional annotation of transcriptomes

**Figure 1:**
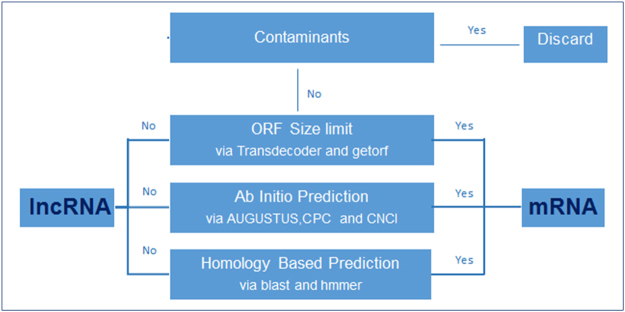
The pipeline for the identification and annotation of both coding transcripts and lncRNAs. Before the identification of both coding transcripts and lncRNAs, the organellar transcripts and small RNAs were eliminated from the transcripts which were all together named as ‘contaminants’. The open reading frame (ORF) of transcripts were detected with two different softwares, Transdecoder and EMBOSS:getorf, since the presence of functional ORF is the most important criteria for identification of both coding transcripts and lncRNAs. Coding potential of transcripts was calculated with two different programs, CNCI and CPC, in addition to the online gene prediction tool, AUGUSTUS. Additionally, the homology between transcripts and known proteins sequences were inspected, together with the functional protein domains.

Gene content of each transcriptome assembly was evaluated through four layers of analyses as described in Fig. 1. All transcripts were initially screened against known small non-coding RNA sequences and mitochondria/chloroplast originated sequences of *Triticum* families with Blast tool kit^28^. Overall, less than %1 of transcripts with significant hits in these screenings were considered as contaminants and excluded from the Kiziltan, TR39477 and TTD-22 transcriptome assemblies. Subsequent to contaminant analysis, open reading frame (ORF) content of the remaining transcripts was analyzed and transcripts possess ORFs longer than 80 aa were further evaluated for their coding potential through *ab initio* techniques; CPC^29^, CNCI^30^ and AUGUSTUS^31^. Totally, 60% of Kiziltan, 62% of TR39477 and 64% of TTD-22 transcriptome showed coding potential evidence, respectively. These transcripts were further evaluated for detection of their homology to known protein sequences and/or presence of functional protein domains. Ultimately, a total of 84,288, 75,996 and 78,456 putative protein-coding transcripts were identified from Kiziltan, TR39477 and TTD-22, which corresponded to 35%, 37% and 39% of the assemblies, respectively.

Although the assemblies were constructed with the utilization of data from pooled samples of three different biological replicate for each variety, assembled contigs might show none-to-very low expression level; thus, identification of actively-expressed transcripts is necessary for further characterization of transcriptomic data. To determine expression levels of transcripts, transcript abundances were quantified in terms of Fragment Per Kilobase Million mapped reads (FPKM) using RSEM package under Trinity software. Expression activity of protein-coding transcripts was evaluated based on the normalized FPKM. Percent of transcripts that failed to satisfy FPKM cutoffs in both control and drought stressed samples were plotted over a range of FPKM thresholds (Supplementary Fig. 1). Overall, 1% change observed between five cut-offs from 0.1 to 0.5 FPKM; however, a sudden 1% change occurred thereafter. The point, 0.5 FPKM, was chosen arbitrarily as this was the point where the slope of the curve changes, indicating the significance of this point, thereby suggesting it as a potential threshold. With this threshold, 95% of each transcriptome were found to be actively-expressed transcripts, indicating the quality of the transcriptome assemblies and good coverage of the sequencing. In total, 81,168 (96%), 73,465 (97%) and 75,861 (97%) actively-expressed protein-coding transcripts (called coding transcript from this point) were identified in Kiziltan, TR39477 and TTD-22, respectively (Supplementary File 1).

The actively-expressed coding transcripts were inspected for their expression patterns in control and drought treated samples. All three *T. turgidum* varieties represented a high portion of common transcripts between controlled and stressed samples where more than 70% of transcripts were detected as common (60,520; 57,012 and 56,164 transcripts for Kiziltan, TR39477 and TTD-22, respectively). Sample specific transcripts were most abundant in control samples, where 14,595, 10,865 and 14,204 transcripts expressed from solely controlled Kiziltan, TR39477 and TTD-22 varieties, respectively, as opposed to that of 7% of transcripts (6,053; 5,588 and 5,493 transcripts for Kiziltan, TR39477 and TTD-22, respectively) expressed only drought-stressed samples. Blast alignments of drought-specific transcripts revealed that 1,034 homologous transcripts (>80% of query identity and coverage) expressed in both tolerant and susceptible varieties. Drought-tolerant TR39477 revealed 36 different transcripts which does not have any similarity to transcripts from drought-susceptible TTD-22 while 4 of the TTD-22 transcripts were detected as TTD-specific (Supplementary Table 1). These transcripts were remarked as effective on the different drought stress tolerance and adaptation mechanism of these wheat varieties.

Functional annotation of all coding transcripts was conducted by Gene Ontology (GO) term assignment followed with KEGG pathway and COG analysis via Blast2GO software^32^. In total, 860,446 GO terms were assigned to 157,013 (68% of all coding transcripts) transcripts. Assigned GO annotations were clustered in three main categories; Molecular Function (MF), Cellular Component (CC) and Biological Process (BP), based on the blast hits from the NCBI non-redundant (nr) *Viridiplantae* protein database with an e-value cutoff of 1E-5. Across all GO annotations, ‘ATP binding’, ‘membrane’ and ‘protein phosphorylation’ were predominant in the MF, CC and BP categories, respectively. Since these included coding transcripts from both treated and untreated samples in the three varieties, the categories most represented by transcripts could be considered as representatives of housekeeping genes. These sequences were further inspected in terms of the blast hit distribution through different plants which revealed the homology pattern of coding transcripts of *T. turgidum* varieties (Supplementary Fig. 2). Blast hit distributions showed that *T. turgidum* coding sequences possess the highest homology with *Aegilops tauschii* sequences where 26% of transcript revealed as identical to proteins from this species, followed by *Triticum Urartu* (24%) and *Hordeum vulgare ssp. vulgare* (24%) (Supplementary Fig. 2A). Additionally, e-value distribution of blast top hits indicated the general quality of the assembled coding transcripts where more than 50% of the hits have e-values smaller than 1e-110 (Supplementary Fig. 2B). Following Blast2GO annotations, mapping against KEGG database were performed to retain relative biological pathways of the coding transcripts^33^. In total, 50,250 transcripts were assigned to a total of 133 pathways in KEGG database. KEGG pathways the most represented by transcripts were purine metabolism (7,799 transcripts, 15.5%), thiamine metabolism (6,302 transcripts, 12.5%) and biosynthesis of antibiotics (3,147 transcripts, 6.3%) across all transcripts. Additionally, Cluster of Orthologous Groups (COG) screenings were performed using EggNog database, under Blast2GO software^34^ and coding transcripts sharing similar functions were classified into 23 functional groups. The largest group represented by transcripts had functions defined as ‘unknown’ (5,935 transcripts, 23.5%) followed by ‘posttranslational modification, protein turnover and chaperones’ (2,545 transcripts, 10.1%), ‘signal transduction mechanisms’ (2,397 transcripts, 9.5%), ‘intracellular trafficking, secretion and vesicular transport’ (1,600 transcripts, 6.3%) and ‘translation, ribosomal structure and biogenesis’ (1,506 transcripts, 6%).

**Figure 2:**
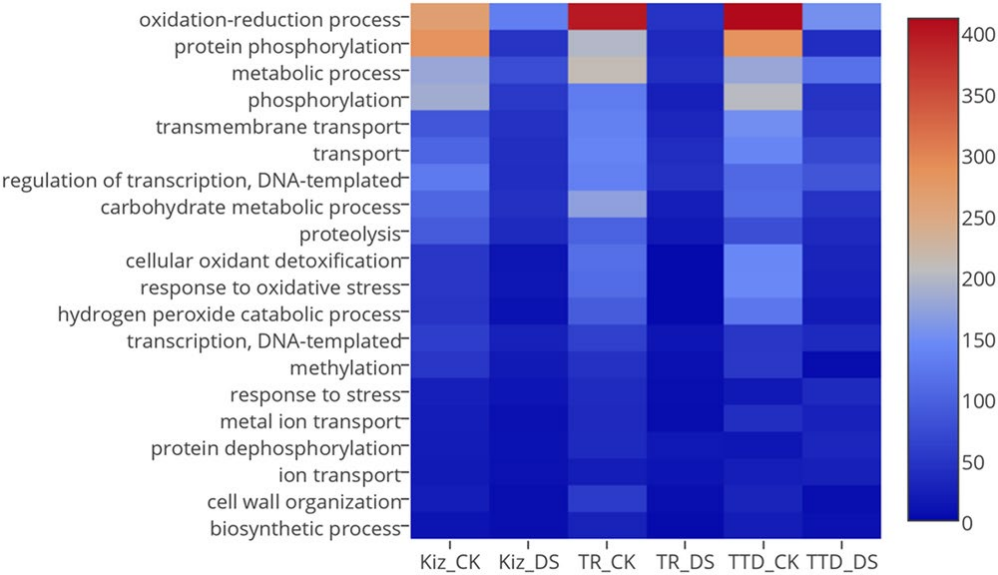
Heatmap for top 20 biological processes represented by stress-responsive coding transcripts in each sample. Several biological processed were detected and drought treated samples. (Graph legend: Kiz_CK: mRNAs from variety Kiziltan expressed under control conditions, Kiz_DS: mRNAs from variety Kiziltan expressed under drought stress, TR_CK: mRNAs from variety TR39477 expressed under control conditions, TR_DS: mRNAs from variety TR39477 expressed under drought stress, TTD_CK: mRNAs from variety TTD-22 expressed under control conditions, TTD_DS: mRNAs from variety TTD-22 expressed under drought stress.

The functions of differentially expressed transcripts were further analyzed after the annotation of all transcripts. In all the three plants, ‘oxidation-reduction’ and ‘protein phosphorylation’ were the most represented BP terms in transcripts exhibited differential expression in response to drought stress (Fig. 2). Interestingly, drought-susceptible TTD-22 revealed an increased number of upregulated genes which were categorized in ‘response to stress’ group regarding to BP assessment of Blast2GO (from 17 to 37 transcripts) while this category represented less number of associate transcripts in Kiziltan (from 24 to 13 transcripts) and TR39477 (from 37 to 4 transcripts) under drought stress. The orthologous groups of drought specific transcripts were also analyzed to determine their functional importance. The most representative COG id by all the drought specific transcripts, KOG0987, was associated with a DNA helicase which is functional in cell cycle control and cell division. Another important one, COG0507, connected with ‘exodeoxyribonuclease v alpha’ protein involving in replication and recombination. In order to further understand the drought tolerance of TR39477, the unique transcripts which are specifically expressed under drought stress, 36 transcripts which do not show any resemblance to TTD-22 transcripts, were analyzed in regards to their associated KEGG pathway. Mostly, pathways associated with secondary metabolite synthesis were detected as enriched by these unique transcripts such as ‘Glutathione, Sphingolipid and Thiamine metabolism’. Also, ‘Glycosaminoglycan and glycan degradation pathways’ were enhanced by unique transcripts of TR39477. Accordingly, identification and annotation of all such transcripts provided insides regarding drought-responsive metabolomic changes in durum wheat together with their transcript partners.

### Putative lncRNAs and their expression pattern under drought stress

**Figure 3:**
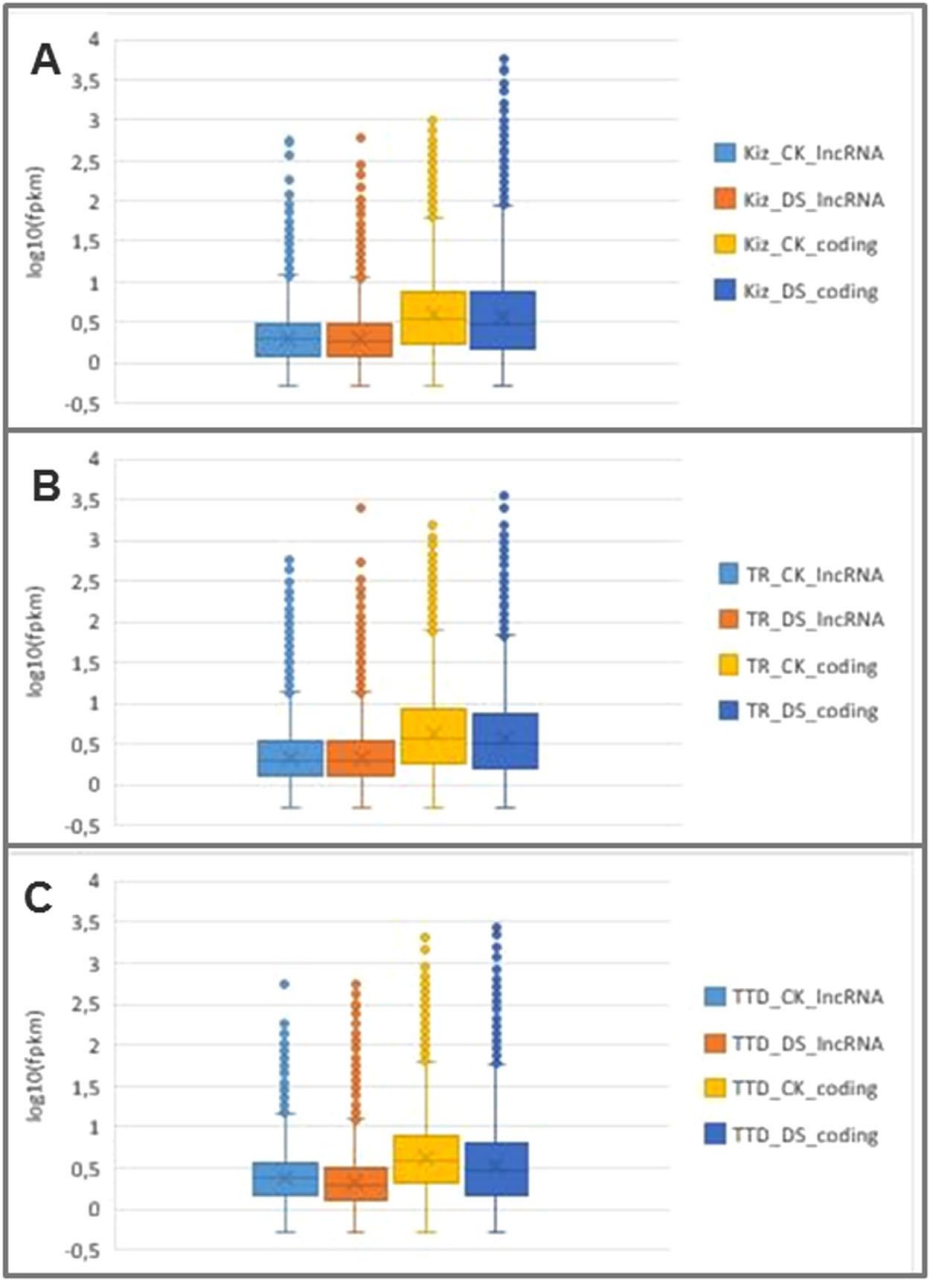
Expression pattern of coding transcripts and lncRNAs in three different *T. turgidum* samples. (**A**) The expression pattern of coding transcripts and lncRNAs from Kiziltan variety which exhibit moderate performance under drought conditions. (**B**) The expression pattern of coding transcripts and lncRNAs from drought tolerant TR39477 variety. (**C**) The expression pattern of coding transcripts and lncRNAs from drought susceptible TTD-22 variety.

Identification of long-noncoding RNAs was performed by following the pipeline illustrated in Fig. 1. Totally; 26% (63,773 transcripts), 29% (61,823 transcripts) and 22% (43,932 transcripts) of the transcriptome assemblies from Kiziltan, TR39477 and TTD-22 varieties, respectively, were associated with lncRNAs. Subsequent to identification, the actively-expressed putative lncRNAs were inspected based on normalized FPKM value obtained from transcripts abundance estimation analysis and total of 59,110 (93%), 57,944 (94%) and 40,858 (93%) putative lncRNAs were identified as actively-expressed putative lncRNAs (called lncRNAs from now on) from Kiziltan, TR39477 and TTD-22 varieties, respectively. The slightly lower ratio of active expression in lncRNAs (93–94%) compared to coding transcripts (96–97%) might arise from the tendency of lower expression of lncRNAs. Additionally, inspection of the expression patterns of transcripts in each control and drought-stressed samples revealed a similar distribution of the expressions of the lncRNA and coding transcripts between the three biological replicates only with little systematic biases (Fig. 3). Error plots showed lower expression levels of lncRNAs compared to coding transcripts across all three plants which is also supported by literature^21^. Moreover, overall expression pattern of lncRNAs were not altered by drought treatment, except a few transcripts.

**Figure 4:**
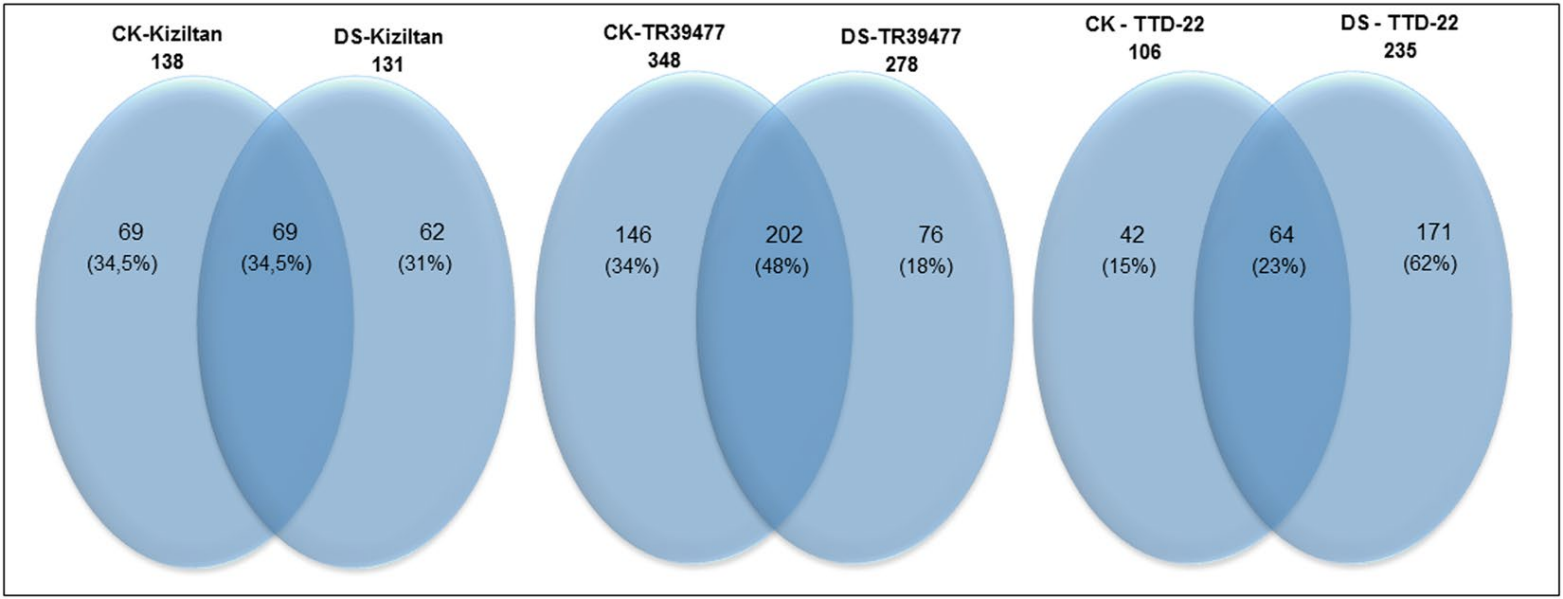
Common and drought specific lncRNAs from different *T. turgidum* varieties. Venn diagrams show the number of common and specific differentially expressed lncRNAs between control (CK) and drought-stressed (DS) samples of Kiziltan, TR39477 and TTD-22.

Several lncRNAs from three different plants showed differential expression under drought treatment. These lncRNAs were identified with edgeR software with a p-value smaller than 0,001 and log2 (fold change) greater than 2^35^. Based on these cut-offs, 200 (3% of all lncRNAs), 424 (6% of all lncRNAs) and 277 (4% of all lncRNAs) were detected as ‘drought-responsive’ from Kiziltan, TR39477 and TTD-22, respectively. Differentially expressed lncRNAs were further evaluated for their sample specific expressions. Most of the differentially expressed lncRNAs showed tendency to exhibit sample specific expressions, indicating distinct molecular functions they might perform. Intriguingly, 66, 52 and 77% of differentially expressed lncRNAs exhibited sample specific expressions in Kiziltan, TR39477 and TTD-22 samples, respectively (Fig. 4). From 64 to 202 differentially expressed lncRNAs were detected as common between control and drought treated samples of the three *T. turgidum* plants. In Kiziltan, 35% of stress-responsive lncRNAs were common whereas 48% and 23% stress-responsive lncRNAs were common between control and drought treated samples of TR39477 and TTD-22, respectively. These results indicate that common transcripts were more abundant in differentially-expressed lncRNAs from TR39477, with the most tolerant profile, than Kiziltan, with moderate reaction, and TTD-22, with the least tolerance. However, further characterizations are required for a complete understanding of the lncRNAs functions under drought stress.

**Figure 5:**
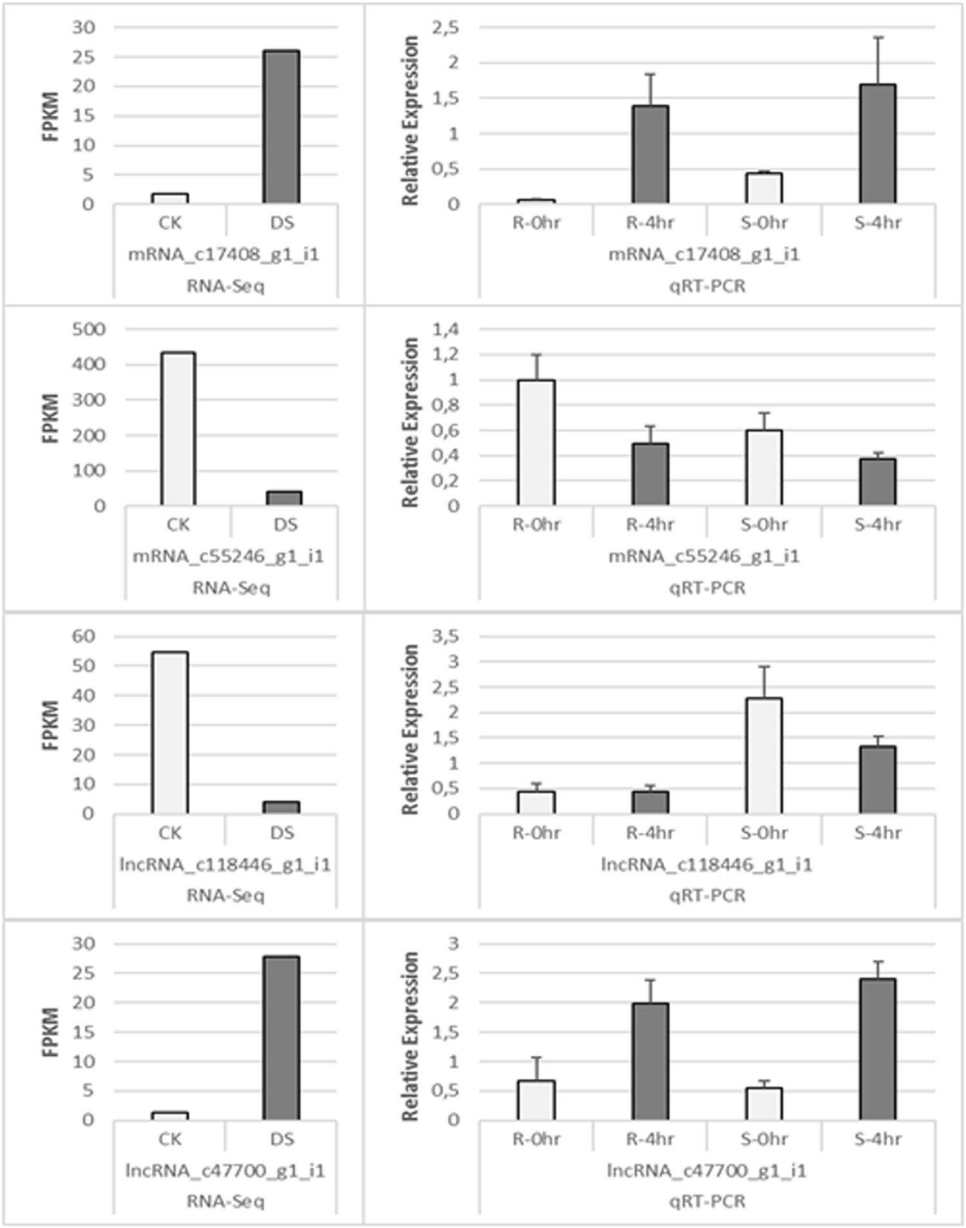
Relative normalized expression analysis results for common differentially expressed mRNA and lncRNAs samples. The quantification of transcript expression was performed with both root and whole-seedling tissue. The error bars were constructed based on standard deviation across three replicates of each sample.

Differential expression of mRNAs and lncRNAs were also confirmed with Quantitative Real Time (qRT-PCR) experiment. Common mRNA and lncRNAs transcripts; defined with %80 identity and query coverage across whole samples; were analyzed and a group of differentially expressed ‘common’ transcripts was chosen for experimental conformation. From this pool, expression of randomly chosen 2 mRNA and 2 lncRNA transcripts were quantified followed by 4 hours of shock drought treatment with 2-week-old root and whole seedling tissues of Kiziltan genotype. The quantification results with QRT-PCR experiment showed accordance with RNA sequencing differential expression data analysis both for lncRNAs and mRNAs (Fig. 5). In addition, experimental results showed harmony between root and whole seedling tissues for lncRNAs and mRNAs.

### Characteristics of actively expressed lncRNAs

All actively expressed lncRNAs were blasted against lncRNAs in *A. thaliana* from NONCODE database^36^. We identified 32, 24 and 15 lncRNAs that were homologous to those lncRNAs in *A. thaliana*, suggesting the weak conservation of lncRNAs between *A. thaliana* and *T. turgidum* species. Actively expressed lncRNAs were further analyzed for their structural features in wild relatives of wheat. To that end, the length distribution and GC content of expressed lncRNAs and coding transcripts were analyzed and compared. The average length of *T. turgidum* lncRNAs was 327 nucleotides long whereas that of coding transcripts was 1,198 nucleotides. Lengths of those lncRNAs ranged from 201 to 2,686 nt, 2,857 nt and 2,540 nt in Kiziltan, TR39477 and TTD-22, respectively. In general, the majority of lncRNAs were relatively short while almost half (47–50%) of coding transcripts were longer than 1,000 nt in all the *T. turgidum* varieties (Supplementary Fig. 3). The average GC content for lncRNAs was ranging from 43% to 45% and across all three varieties, the highest ratio of GC content was 82%. Interestingly, highest GC content of lncRNAs was detected in shorter lncRNAs, generally shorter than 1,000 nt which suggest an association between GC content and lncRNAs length (Supplementary Fig. 4). On the other hand, the average GC content of coding transcripts was detected as 52%, relatively higher than lncRNAs in all three varieties. Connection between GC content and length distribution was also observed in coding transcripts (Supplementary Fig. 4). While the length of the coding transcripts is increasing, the GC content was detected as narrowing around 50%. Overall, these results indicate that lncRNAs as well as coding transcripts share similar structural features in different *T. turgidum* species; yet, lncRNAs slightly differ from coding transcripts in gene structure in terms of structural characteristics.

Tetraploid durum wheat and wild emmer wheat genomes are derived from hybridization of A and B sub-genomes, each contributing to the composition of coding and lncRNA transcripts equally. Since reference genomes for Kiziltan, TR39477 and TTD-22 varieties are not available yet, we analyzed composition of coding and lncRNA transcripts from the recently published assembly of Zavitan (*T. dicoccoides* variety) genome^37^. Using GMAP, we were able to map 89% of the lncRNA transcripts and 97% of coding transcripts to Zavitan genome. As Zavitan is a different cultivar from our three genotypes, we can expect cultivar dependent lncRNAs, resulting in the slight lower ratio of mapped transcripts of lncRNAs. Among these alignments, 2% of coding and 3% of lncRNA transcripts were mapped to uncharacterized scaffolds. On average, 50 and 48% of coding transcripts and 47 and 50% of lncRNA transcripts were mapped to A and B sub-genomes, respectively. 48, 53 and 49% of coding transcripts and 46, 50 and 45% of lncRNA transcripts were mapped to A sub-genome whereas 50, 45 and 50% of coding transcripts and 51, 48 and 52% of lncRNA transcripts were mapped to B sub-genome in Kiziltan, TR39477 and TTD-22 varieties, respectively. The results showed enrichment of both coding and lncRNA transcripts in A sub-genome in TR39477 varieties and in B sub-genomes in Kiziltan and TTD-22 varieties. Both coding and lncRNA transcripts were similarly distributed over each chromosome at frequencies varied between 6 to 9%. These transcripts were most abundant at 2A chromosome for TR39477 and at 2B chromosome for Kiziltan and TTD-22 varieties. The results showed each sub-genome and chromosome of tetraploid wheat genome contributed in composition of lncRNAs as in case of coding transcripts.

One transcript can be derived from different loci and from opposite directions on the genome. The results showed similar distribution of coding and lncRNA transcripts on sense and antisense strands. Nearly 24% of all transcripts were shown to be transcribed from both directions whereas remaining alignments were distributed equally on sense and antisense strands. Consistent with previous studies on plants^38^, most of the lncRNAs (80%) were single-exon transcripts and 6% of lncRNAs could be transcribed as both single-exon and multi-exons transcripts from different loci. On the other hand, coding transcripts tended to have more exons where 76% of coding transcripts transcribed with multi-exons. lncRNA transcripts showed smaller number of exons where maximum exon number can reach up to 16 in a lncRNA transcript as opposed to that of 68 for coding transcripts.

Similar to protein coding transcripts, lncRNAs are also exposed to alternative splicing with a lower rate compared to mRNAs^39^. Trinity-constructed isoforms of each gene were accounted for the spliced isoforms and were used to determine alternative splicing ratios of lncRNAs. In Kiziltan, 18% (10,369) of actively expressed lncRNAs were exposed to alternatively splicing where this ratio was detected as 64% (51,634) for that of coding transcripts. Similarly, alternatively spliced lncRNAs were counted as 18% (10,611) and 16% (6,906) of total lncRNAs where that of 60% (45,138) and 59% (44,221) of coding transcripts were identified as alternatively spliced, in TR39477 and TTD-22, respectively. Furthermore, alternative splicing (AS) events were identified from all mapped transcripts to Zavitan genome. AS events occurred in ~14% of all actively expressed transcripts in each genome. Among the AS events, intron retention with 38% of the events is the predominant over remaining splicing events, followed by 29% to other events, 15% to alternative acceptor, 10% to alternative donor and 8% to exon skipping. Among the transcripts, 25, 22 and 21% of coding transcripts and 5, 5, 4% of lncRNA transcripts were involved in an AS event in Kiziltan, TR39477 and TTD-22 varieties, respectively. Consistent alternative splicing patterns in different *T. turgidum* varieties suggested that alternative splicing is not as prevalent in lncRNAs as it is in coding transcripts.

Among the lncRNAs of trinity-constructed isoforms, the ones with the most abundant splicing events were further inspected. For example, the two lncRNAs, Kiz_both_c65078_g3_i12 and Kiz_both_c65078_g3_i4, were detected as the isoforms of the same gene, Kiz_c65078_g3 which possessed 23 alternatively spliced isoforms in Kiziltan transcriptome. It was also noted that 8 isoforms of this gene showed sample specific expression, where one (Kiz_CK_c65078_g3_i21) of them identified as ‘coding transcript’ in the control sample. In TR39477 transcriptome, maximum number of gene isoforms was observed as 27 for the gene TR_c63034_g2. Among these isoforms, six of them were detected as actively expressed lncRNAs where only two of these lncRNAs showed differential expression during drought treatment. For TTD-22 transcriptome, the gene TTD_c62818_g1 had the most splicing events with 24 isoforms where six and three of them were identified as lncRNAs and coding transcripts, respectively. None of these isoforms showed differential expression during drought treatment. Yet, all lncRNA isoforms exhibited sample specific expressions where none of them were in common between control and drought treated samples. Since expression levels and significance in p-values were low, these sample specific expressions were not defined as differential expression. Regarding to observed alternative splicing patterns, it is tempting to speculate that each alternatively spliced isoforms have different expression profiles and might have differential functions during stress response.

**Figure 6:**
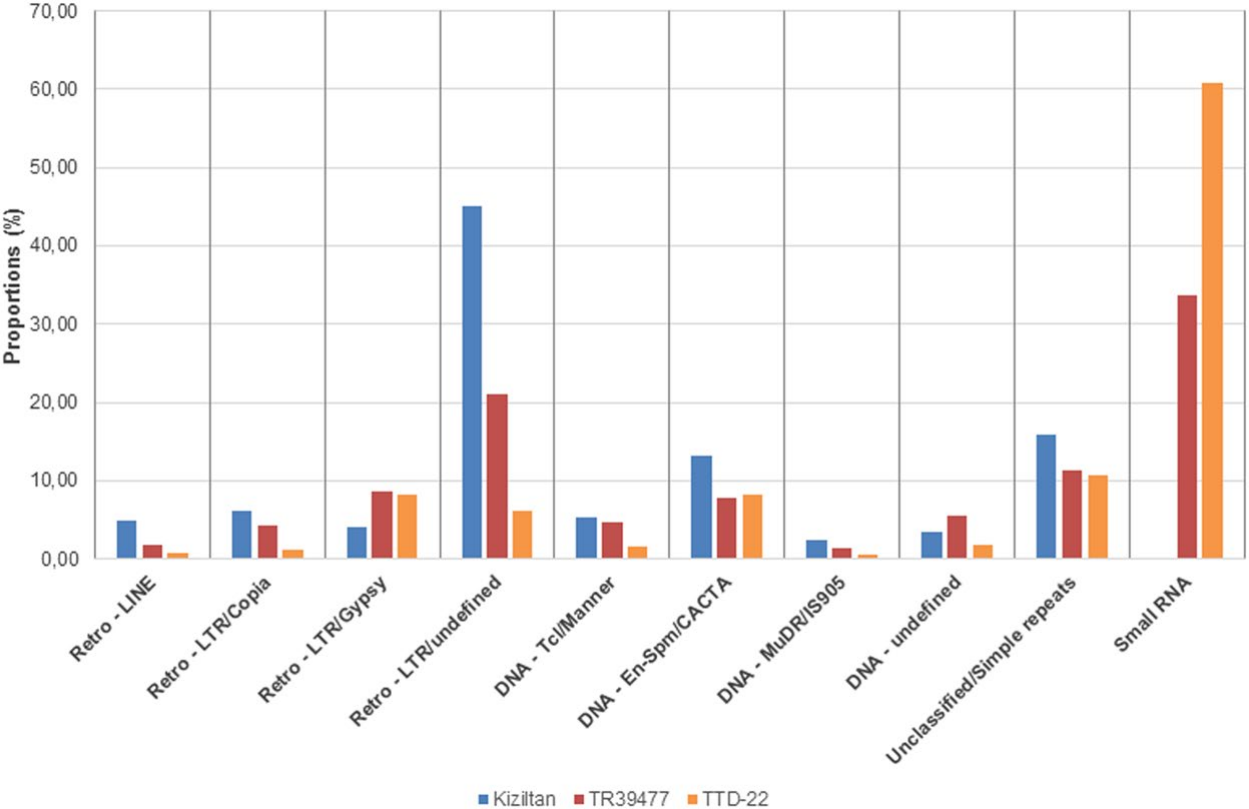
Repeat content of stress-responsive lncRNAs. Stress responsive lncRNAs were associated with both DNA transposons and retrotransposons. Each variety was represented with a different color (blue: Kiziltan, pink: TR39477 and orange: TTD-22).

Repeat-masking of stress-responsive lncRNAs against known Poaceae repeat elements revealed that 37% to 64% of stress-responsive lncRNA sequences contain repetitive elements in three of the replicates. The difference in the repeat content of stress-responsive lncRNAs stem from repeat elements from small RNAs found in *T. dicoccoides* varieties. Once small RNAs excluded from repeat library, percent of stress-responsive lncRNAs containing repeat elements were decreased to 33–34% in both TR39477 and TTD-22 varieties. Interestingly, lncRNAs which were from small RNA sequences, made up 34% or 61% of all repeats (Fig. 6), were detected in *T. dicoccoides* samples, TR39477 and TTD-22 whereas no siRNAs were detected in Kiziltan. These small RNA repeats were from closely related species including *Zea mays, Triticum aestivum* and *Oryza sativa*. The most common small RNA sequences found in both TR39477 and TTD-22 samples was ZRSiRGRR00000035, following ZRSiRGRR00000042 and TRSiRGRR00000062. These observations indicate that some lncRNAs involved in stress response may act as siRNA precursors. Besides, their corresponding siRNAs were, therefore, regulated in a stress dependent manner. Excluding small RNAs from repeat content, stress-responsive lncRNAs which were from DNA transposons were marked in all three samples in almost half percent of repeats.

### miRNA-related functions of lncRNAs

miRNAs can regulate gene expression at the post-transcriptional level by interacting with the complementary binding sites on target sequences, resulting in cleavage, decoy, or translation repression^8, 13, 40^. Several studies have suggested that lncRNAs might have functions associated with miRNAs being either their targets or precursors^19^. To explore such functional roles of lncRNAs, *in silico* miRNA prediction was performed from all of three varieties by utilizing a list of 1,404 high confidence and/or experimentally verified plant miRNAs subtracted from miRBase release 21^41^. In silico miRNA identification process led to the identification of 54, 58 and 46 lncRNAs in Kiziltan, TR39477 and TTD-22, respectively, as putative precursors of miRNAs belonging to 38 miRNA families. Interestingly, only one of the precursor lncRNAs in each assembly exhibited differential expression during drought treatment. In TR39477 and TTD-22, the stress responsive lncRNAs TR_c65168_g7_i1 and TTD_c34631_g1_i1 were detected as the precursors of miR1127 which do not have any determined target in these transcriptome assemblies. On the other hand, in Kiziltan, the stress-responsive lncRNA, Kiz_c66393_g4_i7, was identified as the putative precursor of the two miRNAs, with 1 or 2 nt changes in mature miRNA sequences of *Triticum aestivum* miRNAs; miR1117 and miR1127a. LncRNAs which have ability to generate miRNA sequences might perform an indirect regulatory function through lncRNAs - generated miRNAs. In order to determine this indirect regulatory path, target transcripts of lncRNAs-derived miRNAs were analyzed and targets were identified only for miR1117. miR1117 was associated with one coding; Kiz_c69869_g4_i1: a coding transcript expressed in both control and drought-treated samples without any change in expression; and two noncoding RNA targets; drought-specific Kiz_c106327_g1_i1 and control specific Kiz_c85253_g1_i1. This indicates that lncRNA-derived miRNAs can perform multiple targeting potential which includes both coding and noncoding transcripts, indicating the complex regulatory mechanisms through noncoding RNAs even though the underlying regulatory network is not completely understood. Moreover, differential expression of precursor transcripts might result in the differential expression of corresponding mature miRNAs, leading to an increased regulation of expression; however, analysis of mature miRNAs at small RNA level is necessary for further validation of differential miRNA expression.

In order to provide more insight into miRNA-lncRNA association, functions of lncRNAs were analyzed in the sense of acting as miRNA targets using psRNATarget webtool at the default settings. It was shown that 1,276 lncRNAs were targeted by 33 miRNAs in Kiziltan where 1,124 lncRNAs targeted by 24 miRNAs in TR39477 and 560 lncRNAs by 26 miRNAs in TTD-22. In Kiziltan, 9 of the lncRNAs targeted by miRNAs, further suggesting 13 stress-responsive miRNA-lncRNA target pairs, detected as differentially expressed in drought condition (Supplementary Table 2). In TR39477, 15 stress-responsive lncRNAs were detected as putative miRNA targets, building 27 unique miRNA-lncRNA target pairs (Supplementary File 2). Yet, only 4 of the target lncRNA transcripts that established 7 miRNA-lncRNA target pairs in TTD-22 showed differential expression between drought-stressed and control samples (Supplementary File 2). Intriguingly, miRNAs targetting stress-responsive lncRNAs were mostly dominated by miR1436 and miR1439, where miR1118, miR1122, miR1130, miR1137 and miR1139 possessed putative lncRNA targets in TR39477 samples only, and miR1133 and miR1136 targeted lncRNAs in Kiziltan and TR39477, moderate to high tolerant samples (Supplemental File 2). Additionally, it was shown that, in accordance with drought tolerance profiles of the samples, miR1436 and miR1439 mediated 8, 16 and 5 miRNA-lncRNA target pairs in Kiziltan, TR39477 and TTD-22 samples, respectively. These results suggested that gene regulation of miRNAs on stress-responsive lncRNAs are well corralated with the stress-tolerance profiles of the three genotypes such that stress-responsive miRNA-lncRNA target pairs were prevalent at most in TR39477, the most tolerant genotype and vice versa in TTD-22, the most sensitive genotype to drought. Moreover, the diversity of target lncRNAs in the most tolerant variety, TR39477, might be an indicator of additional regulatory mechanisms mediated by these lncRNAs. Thus, functional characterization of these target lncRNAs may shed light onto the drought tolerance mechanisms in *T. turgidum* species.

### Functional characterization of lncRNAs through lncRNA-miRNA-mRNA networks

**Figure 7:**
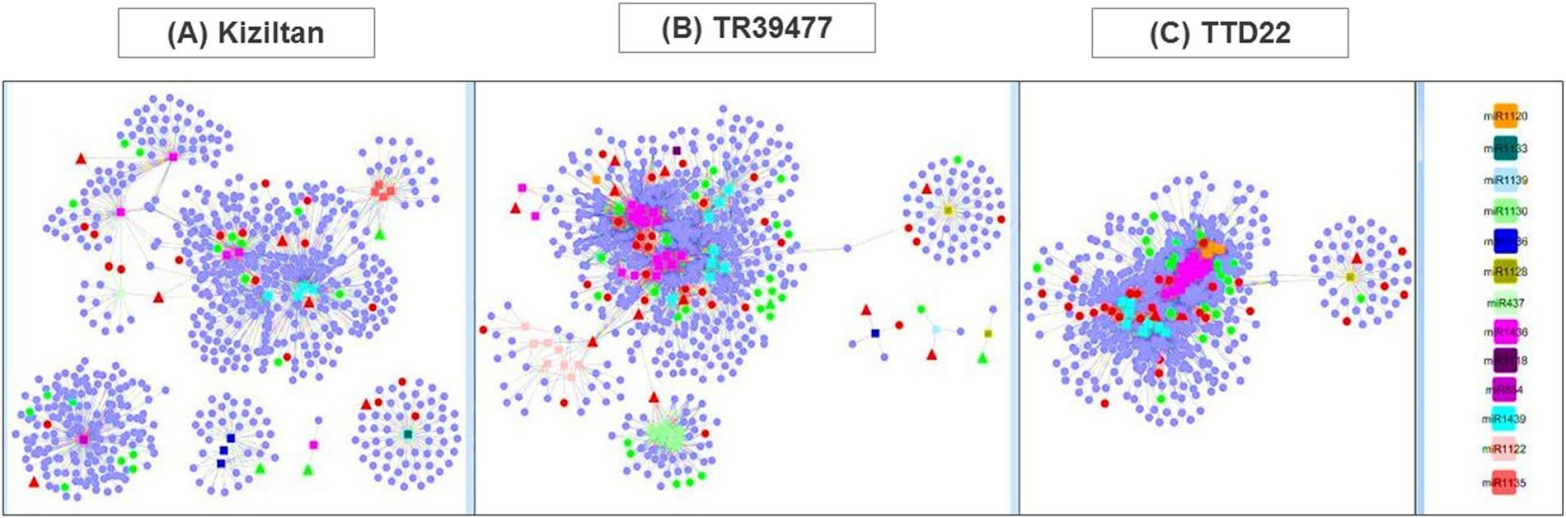
miRNA regulated networks between lncRNAs and coding transcripts. miRNA-lncRNA and mRNA networks were represented for each variety, Kiziltan (**A**), TR39477 (**B**) and TTD-22 (**C**). miRNA nodes were presented as rectangle and colored by miRNA family names. lncRNAs and coding transcripts were presented as triangles and circles, respectively. Transcripts that were upregulated by drought were colored as red and downregulated transcripts were colored green.

**Figure 8:**
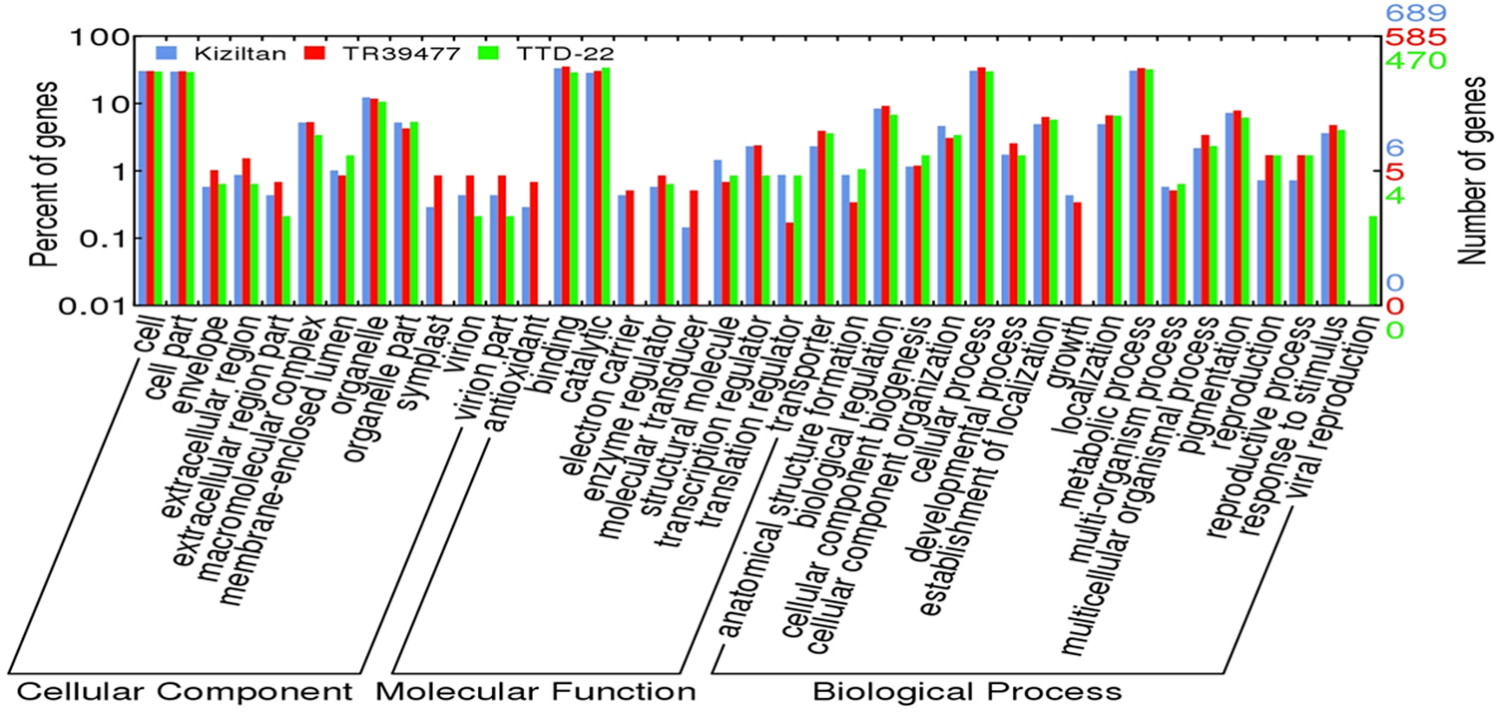
Distribution of Gene Ontology mapping results of coding targets of putative miRNAs. Targets from each variety was represented with a different color; blue: Kiziltan, red:TR39477, green: TTD-22. GO terms histogram was prepared through WEGO online tool.

**Figure 9:**
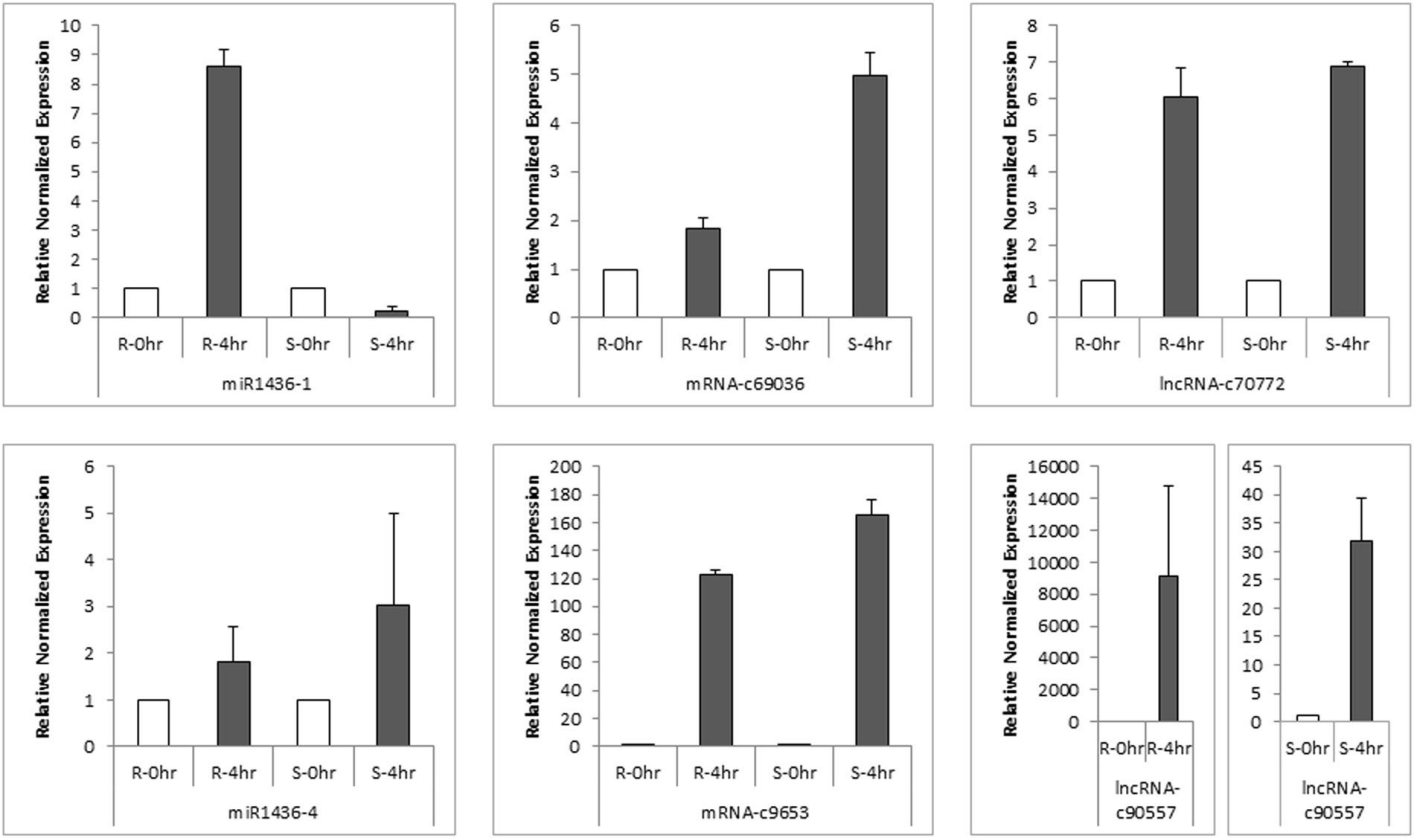
Relative normalized expression analysis results for lncRNA-miRNA-mRNA networks involved differentially expressed transcripts. The quantification of transcript expression was performed with both root and whole-seedling tissue. The error bars were constructed based on standard deviation across three replicates of each sample.

lncRNAs may interrupt miRNA-based regulation of gene expression by target mimicry where miRNAs bind to lncRNAs instead of their actual mRNA targets^42^. Thus, lncRNAs may indirectly enhance functioning of particular coding transcripts by preventing negative regulation of their translation by miRNAs. To explore stress specific association of miRNAs, lncRNAs and mRNAs, stress-responsive lncRNAs and their miRNA-mRNA network was particularly analyzed. lncRNAs-miRNA-mRNA networks were observed in all three varieties with different levels of complexities (Fig. 7, Supplementary File 2). The most complex interaction networks were established by miR1436 and miR1439. Intriguingly, these two miRNAs were detected as sharing similar target transcripts in all samples, indicating a dual-regulation of gene expression. Besides it was shown that miR437 and miR1135; miR1120, miR1122, miR1128 and miR1130; and miR1120 and miR1128 were also contributing to the interaction circuitry of miR1436 and miR1439 in Kiziltan, TR39477 and TTD-22 samples, respectively, suggesting additional players in these complex networks. Among these, interactions through miR1120 and miR1128 were conserved at the interspecies level. The putative functions of coding transcripts were elucidated through GO mapping annotations. Intriguingly, antioxidant, electron carrier and molecular transducer molecular functions and growth biological process were highly enriched in Kiziltan and TR39477 varieties but no evidence was found in TTD-22 varieties (Fig. 8). These results suggested that increased regulation in these functions might be involved in drought stress response in *T. turgidum* varieties. Among the interaction networks, expression profiles of miRNAs (mir1436–1 and miR1436–4) and their corresponding lncRNA (c70772_g2_i1 and c90557_g1_i1) and mRNA (c69036_g1_i1 and c9653_g1_i2) targets were shown in Fig. 9. The expression of mRNA and lncRNAs molecules were concordant with the RNA-Seq data where both mRNAs and lncRNAs are upregulated under drought stress. Additionally, Q-RT-PCR results proved the drought specific expression of lncRNA c90557_g1_i1 where no expression for this lncRNAs was detected under control condition in variety Kiziltan.

## Discussion

The increased effects of drought stress, caused by climate change, compel the improvement of major crop species such as wheat. However, complex genome of hexaploid wheat, combined from three different sub-genomes, A, B and D, becloud the understanding of gene regulations and molecular pathways underlying stress adaptation mechanisms, which is essential for establishing better crop performance. Alternatively, tetraploid wheat species, possessing a less complex genomic organization, stand as good candidates to pave the way for a deeper understanding of such mechanisms in wheat. Several varieties of wild tetraploid wheat have already been shown to exhibit differential drought tolerance, which might enhance our understanding of the drought-tolerance mechanisms in bread wheat^9^. With the aim of providing further insights to drought response mechanisms and associated stress tolerance profiles of tetraploid wheat, transcriptomic changes in the roots of three different T. turgidum samples under slow drought imposition were analyzed at both coding and non-coding levels. Overall, this study showed the differential regulation of both coding and non-coding transcripts in response to drought stress, which might further be used for a better crop performance under drought conditions.

Sequenced reads from both control and drought-treated samples were assembled together and analyzed in the sense of differentially expressed coding transcripts and lncRNAs. A stringent filtering of transcripts (Fig. 1) enabled the identification of 35, 36 and 39% of actively-expressed coding transcripts besides 26, 29 and 21% of actively-expressed lncRNAs over all actively-expressed transcripts in Kiziltan, TR39477 and TTD-22 varieties, respectively (Supplementary Table 3). Overall, 2 mRNA and 2 lncRNA transcripts were validated with qRT-PCR experiment and the expression trend of transcript showed a similar pattern with RNA-Seq data analysis even though the fold changes are different. Since the shock drought stress treatment was used for validation of presence of these transcripts, this is an expected result; particularly for lncRNAs, which the expression is highly dependent on condition. In the RNA-Seq analysis, interestingly, the number of transcripts was detected as decreased in all three varieties, regardless of their stress tolerance (Table 1). The stress-induced protein breakdown is a known phenomenon in plants where the accumulated amino acids support the osmotic balances in cells^43^. Thus, it might be possible that the number of transcripts which leads to translation of several proteins may decrease to further support this breakage and osmotic balance. Additionally, it was noted that although having the highest percent of coding transcripts, the transcriptome of TTD-22, the most sensitive genotype, contained the lowest percent of lncRNAs. As several studies have provided evidence of the functional importance of lncRNAs for drought stress response^44, 45^, the low abundance of lncRNAs in TTD-22 might be associated with its low drought tolerance; however, further characterization of stress responsive lncRNAs, particularly from drought tolerant variety TR39477, is essential to fully understand the role of lncRNAs in drought response.

To understand the function of differentially expressed genes under drought stress, the functional annotation was conducted via BlastX and Blast2GO. Analysis of homology patterns in *T. turgidum* proteins revealed a scattered homology of proteins across different *Poaceae* members. Although a high homology of *T. urartu* proteins is expected because of heritage of tetraploid wheat, high homology with *A. tauchii* stands as unexpected and further examination of these homolog proteins might provide insight into evolution of *T. turgidum*. The conservation of different coding transcripts was also observed between different accessions of *T. turgidum*. Additionally, more than 70% of transcripts were detected as common under control and drought-treated samples. Since plant cells tries to keep basal reaction rate for cellular maintenance, it is normal for high conservation of proteins under drought treatment^46^. Moreover, even though a small portion of transcripts was differentially expressed under drought stress, they might have serious effect on other proteins.

Drought specific transcripts from TR39477 and TTD-22 were further analyzed in regards to their function to further understand the differences in the drought tolerance. Comparison of these transcripts from two varieties revealed that only approximately 20% of these transcripts are conserved. These common transcripts, expressed in response to drought regardless of the degree of drought tolerance, might be associated with general proteins which are expressed in the stress conditions such as ABA-responsive transcription factors of ROS scavengers^43^. On the other hand, TR39477 revealed 36 transcripts which are specific to this cultivar and do not possess any degree of homology to TTD-22 transcripts. These transcripts were connected with several osmolytes and secondary metabolite such as ‘Glutathione’ and ‘Thiamine’ metabolisms regarding to KEGG maps. For instance, glutathione metabolism was associated with proline production, which is an important osmolytes accumulated in drought stress^47^. Thiamine is an important molecule which involves in to phenylpropanoid pathway and this pathway cause the generation of several secondary metabolites which enhance the performance of plants under drought stress^43, 48^. Thus, it is tempting to speculate that TR39477 utilize these transcripts to regulate osmolytes production together with secondary metabolites to survive under drought stress. Further characterization of these transcripts may provide more insights into the molecular mechanisms of these events.

Besides the stress responsive transcripts, lncRNAs exclusive to drought stress and their relationship with miRNAs and mRNAs provided further insight to the molecular mechanism of drought tolerance. The most complex networks were detected for miR1436 and miR1439, which indicates their important function in drought stress response. Among these, miR1439 was detected as targeting an aquaporins proteins which is conserved in wheat, rice and Brachypodium^49^. Under drought stress, lncRNAs might embed the inhibition of aquaporin translation, via target mimicry to miR1439 family members, and enhance the function of this protein for further transport of water from roots. Interestingly, miR1120 and miR1128 detected as conserved at interspecies level suggesting its important function. In another study, Yao and colleagues also detected ubiquitous expression pattern of miR1128 (misnamed as miR504 in the publication) even though no information about the targets of these miRNAs again not suggested^50^. Computational inspection and experimental validation of targets of these miRNAs might shed light onto their presence in these networks. Here, with Q-RT-PCR, we validated expression of miRNA1436-1 and miR1436-4 and their corresponding targets, supporting the existence of lncRNA-miRNA-mRNA networks (Fig. 9).

Gene regulation is not limited to protein-coding genes where most of the genes transcribed in complex organisms are in fact non-protein-coding genes with important regulatory functions. Increasing number of studies has showed that both sRNAs and lncRNAs are important players of gene regulation in various vital biological processes, including stress responses in plants. Drought is a major stress factor to crops, causing serious yield losses to wheat (*Triticum ssp*.), and an important food source worldwide. On top of being an important limiting factor to the yield already, the effect of drought has been expected to increase by climate changes. Improved crop varieties that are tolerant to drought could sustain increased yield and quality of crops. In order to obtain improved varieties with enhanced productivity and stress tolerance, introgression of favorable elements into domesticated crop varieties has been suggested as a viable approach for decades. However, understanding of the molecular mechanisms behind drought response is crucial in determining these elements. The current study provides a comprehensive transcriptome analysis of tetraploid wild wheat varieties with diverse stress tolerance profiles, revealing drought-responsive genes and lncRNAs, thereby enriching the genetic information available for *T. turgidum* varieties. Further in silico predictions of miRNAs and their target interactions exploited the putative functional roles of lncRNAs. Besides, identification and characterization of lncRNAs in the present study expands the current knowledge of lncRNAs and their regulatory roles in drought response in plants in general.

## Methods

### Total transcriptome sequencing, assembly and identification of differentially expressed transcripts

In a previous study of our group, a number of wild wheat varieties were subjected to slow drought imposition where two wild emmer wheat (*T. turgidum ssp. dicoccoides*) varieties, TR39477 and TTD-22, exhibited contrasting responses as the most tolerant and the most sensitive compared to the cultivated durum wheat (*T. turgidum ssp. durum*) variety Kiziltan with moderate response^8^. Total RNA isolation from a pool of three biological replicates of the root samples of control and drought-stressed modern durum wheat, Kiziltan, and wild emmer wheats, TR39477 and TTD-22, conducted with TRI Reagent (Molecular Research Center, Cincinnati, OH, USA) following the manufacturer’s recommendations and RNA integrity was controlled using Agilent Bioanalyzer 2100 RNA 6000 Nano Kit (Agilent Technologies, Santa Clara, CA, USA^8^. Following, high-throughput sequencing with Illumina HiSeq. 2000 were performed with the libraries prepared by using TruSeq RNA Sample Prep Kit v2 ^8^. Illumina HiSeq. 2000 paired end reads can be accessible at ENA database with the run ID: ERR1987529. Raw paired-end reads from RNA sequencing of these six samples (three genotypes × two conditions) were quality trimmed using Trimmomatic (v0.32) with default parameters (LEADING:5, TRAILING:5, MINLEN:36)^26^. De novo assembly for each genotype was generated by Trinity platform^27^ (release 2014-07-17) from combination of paired-end Illumina reads of control and drought-stressed samples. Assembled transcripts were aligned back to the raw reads using bowtie aligner and the abundance estimation of all transcripts was quantified as FPKM with utilization of RSEM under Trinity pipeline. Individual assembly files for each control and drought-stressed samples were separated based on their corresponding abundance estimates for further analysis. Differential expression analysis was conducted using EdgeR pipeline﻿﻿^35^ with the default threshold parameters of p-value = 0,001 and log2(fold_change) = 2.

### Annotation of transcripts and identification of long non-coding RNAs

Following transcriptome assembly, annotation of transcripts and identification of lncRNAs were performed through following rigorous criteria: exclusion of contaminants, open reading frame (ORF) size prediction, *ab initio* predictions and homology screenings. As the first layer of analyses, transcripts were excluded from the assemblies if defined as contaminants after blast screenings against *Triticum turgidum* non-coding RNAs deposited at NCBI and ENA databases (1E-05, -pident 95, -length 30); rRNA, tRNA, snoRNA, snRNA sequences of *Triticum* families deposited in NCBI database (1E-05, -pident 95, -length 30); *Triticum aestivum* mitochondrion complete genome (NC_007579.1) and *Triticum turgidum* organellar RNAs deposited at NCBI and ENA databases (1E-15, -pident 95, -length 30). Remaining analyses evaluated the coding potential of transcripts and aid to determine either lncRNAs or coding transcripts.

Subsequent to contaminant analysis, the ORF size prediction for each assembly was conducted in order to differentiate between protein coding and non-coding transcripts. Since many transposons have similar ORFs to host genes which may corrupt the coding gene annotation, all assemblies were subjected to repeat-masking prior to ORF content predictions, against the repeat library of MIPS Repeat Element Database v9.3 p for *Poaceae* (ftp://ftp.mips.helmholz-muenchen.de/plants/REdat/)^51^ using RepeatMasker v4.0.5 software^52^. Ability of repeat-masked transcripts to construct a full-length protein was evaluated by employing two different software, Transdecoder (*-m* 80) and EMBOSS:getorf^53^. Transcripts with a continuous ORF > 240 nucleotide in length were accepted as transcripts with a functional ORF. Coding potentials of transcripts were predicted with several *ab initio* methods; CPC online tool (*options:* reverse strand mode was included)^29^, CNCI (version 2, *options:* -m pl)^30^ and AUGUSTUS online tool^31^ with the pre-established system trained for Triticum/wheat. Transcripts identified as ‘coding’ by at least one of these tools satisfy the *ab initio* prediction criterion.

In order to identify homolog coding transcripts with other species, assemblies were aligned to a dataset of coding sequences using Blast tool kit (version 31)^28^. All transcripts were initially blasted against several datasets; Uniprot/Swissprot database (http://web.expasy.org/docs/swiss-prot_guideline.html) (parameters: *-evalue* 1E-05, *-pident* 80, *-length* 30); *Triticum aestivum* UniGenes (https://www.ncbi.nlm.nih.gov/unigene, build#63) (parameters: *-evalue* 1E-30, *-pident* 98, *-length* 90, *-max-target-seqs*. 1); *Triticum turgidum* ESTs and coding sequences deposited at NCBI (https://www.ncbi.nlm.nih.gov/) and ENA (http://www.ebi.ac.uk/ena) databases (parameters: *-evalue* 1E-05, *-pident* 95, *-length* 30) together with fully annotated proteins from *Brachypodium distachyon* (annotation: v1.2, http://mips.helmholtz-muenchen.de/plant/brachypodium)^54^, *Oryza sativa* (assembly: IRGSP-1.0, http://rapdb.dna.affrc.go.jp(download/irgsp1.html)^55^, *Sorghum bicolor* (annotation: v1.4, http://mips.helmholtz-muenchen.de/plant/sorghum)^56^, high confidence proteins from *Hordeum vulgare* (http://mips.helmholtz-muenchen.de/plant/barley/)^57^, and Triticum UniProt sequences (144,397 entries, http://uniprot.org/) (parameters: *-evalue* 1E-05, *-pident* 95, *-length* 30). Additionally, Transdecoder (*-m* 30) predicted peptide sequences of each transcript were screened against Swissprot entries (parameters: *-blastp*, *-evalue* 1E-05, *-pident* 80, *-length* 30). Conserved protein domains preserved in these peptides were also controlled with Hmmer (v.3.1b1) against Pfam domains (*-evalue* 1E-05)^58^. Transcripts with homology evidence from any of these screenings satisfy homology-based prediction criterion.

As described in Fig. 1, after exclusion of contaminants, transcripts qualifying remaining criteria were defined as coding transcripts where transcripts with no evidence of coding in ORF size predictions, *ab initio* predictions and homology screenings were defined as lncRNAs. Final functional annotation of coding transcripts were carried out using Blast2GO software^32^ with the initial blast screen run locally against all *Viridiplantae* (taxid: 33090) proteins from the NCBI nr protein database (parameters: *blastx*, *-evalue* 1E-5, -*outfmt* 5, *-max_target_seq*. 1).

### Genome mapping and splicing

Wild emmer wheat genome was obtained from WEWseq consortium (Zavitan_v2_pseudomolecule: http://wewseq.wixsite.com/consortium ^37^). Transcripts were mapped to Zavitan genome using GMAP (version 2016–07–11, with all parameters set to default except –min-identity = 90 –cross-species -f 2). Obtained GFF files were converted into GTF format using gffread. GTF files were submitted to ASTALAVISTA database at default settings to identify alternative splicing events.

### Quantitative Real Time PCR (QRT-PCR) analysis for miRNA, mRNA and lncRNA transcripts

In order to show the accordance of differential expression analysis based on RNA sequencing with wet lab, quantitative Real Time PCR (qRT-PCR) experiment was performed. Prior to experiment, a subset of lncRNAs and mRNAs were selected. The differentially expressed transcript across Kiziltan, TR39477 and TTD-22 were compared via blast analysis and transcript sequences showing similarity with 80% identity and query coverage between whole samples was defined as ‘common’. Between common transcripts, 2 mRNA (Kiziltan_ mRNA_c17408_g1_i1, Kiziltan_ mRNA_c55246_g1_i1) and 2 lncRNA sequences (Kiziltan_ lncRNA_c118446_g1_i1 and lncRNA_c47700_g1_i1) were chosen randomly and qRT-PCR primers were designed (Supplementary Table 4). Kiziltan seed were surface sterilized in 4% sodium hypochlorite and grown in tall plastic jars for 15 days at 23 °C with adequate amount of water. At the end of two week, plant seedlings reached the four-leaf stage were dehydration shocked for 4 hours by removing them from plastic jars and leaving on paper towels under the same lighting conditions, while control plants were immediately fast frozen in liquid nitrogen. Both root and whole seedling tissues were collected and stored at −80 °C. Total RNA isolation from whole collected tissues from control and drought treated samples was performed with TRI Reagent (Molecular Research Center, Cincinnati, OH, USA) following the manufacturer’s recommendations. First strand cDNA synthesis was performed on 1 µg of total RNA using RevertAid H Minus Reverse Transcriptase (QuantiTect Reverse Transcription Kit, Qiagen) according to manufacturer’s protocols. For quantification of mRNA and lncRNAs transcripts from control/ drought stressed root/whole seedling samples, the reaction mix containing 3 µl of 5X diluted of cDNA, 1 µl of forward primers, 1 µl of reverse primer and 5 µl of iTaq^TM^ Universal SYBR® Green Supermix (Bio-Rad) incubated in Bio-Rad CFX 96 Thermal Cycler with following conditions: 95 °C for 10 min followed by 40 cycles of 95 °C for 15 s, 60 °C for 1 min and then 95 °C for 15 s. The constitutive gene of *Triticum aestivum* Actin (TaActin)^59^ was used as internal standard to normalize the transcripts using a gene-specific primer (Supplementary Table 4). The 2-ΔCt method was used to calculate the difference in expression of chosen genes^60^.

For validation of mRNA, miRNA and lncRNAs, miR1436-1 and miR1436-4 were chosen and validated together with their mRNA and lncRNA targets. For qRT-PCR analysis of mRNA and lncRNAs, the same method was utilized described in above. In order to obtain cDNA belonging to mature miRNAs, miRNA-specific stem–loop reverse transcription reactions were performed using RevertAid H Minus Reverse Transcriptase with iScript^TM^ Select cDNA Synthesis Kit (BioRad) following the manufacturer’s recommendations with slight modifications. Prior to cDNA synthesis, a mix of 500 ng of DNAase treated RNA and 1 µl of miRNA-specific stem loop PCR was incubated at 65 °C. After incubation, 2 µl of GSP enhancer solution, 4 µl of 5x iScript select reaction mix and 1 µl of iScript reverse transcriptase were added and reaction mix (10 µl) was incubated at 42 °C for 1 hour followed by 5 minutes of 85 °C incubation to heat-inactivate the reverse transcriptase. For validation of miRNA expression, the reaction mix containing 3 µl of 5X diluted of cDNA, 1 µl of forward primers, 1 µl of universal reverse primer and 5 µl of iTaq^TM^ Universal SYBR® Green Supermix (Bio-Rad) incubated in Bio-Rad CFX 96 Thermal Cycler with following conditions: 95 °C for 5 min, followed by 35–45 cycles of 95 °C for 5 s, 60 °C for 10 s, and 72 °C for 1 s. For melting curve analysis, samples were denaturated at 95 °C, then cool to 65 °C at 20 °C per second. The fluorescence signals were collected at 530 nm wavelengthcontinuously from 65 °C to 95 °C at 0.2 °C per second. The constitutive gene of *Triticum aestivum* rRNA26 homolog^61^ was used as internal standard to normalize the miRNA expression (Supplementary Table 4). For internal control, several control genes including TaU6 were attempted and the rRNA26 was chosen because of its expressional stability under different conditions and tissues. The 2-ΔCt method was used to calculate the difference in expression of chosen miRNAs.

### Construction of lncRNA-miRNA-mRNA networks

High confidence and/or experimentally identified mature miRNA sequences from 72 *Viridiplantae* species were collected from miRBase (v21, June 2014)^62^, suggesting a dataset of 1,404 non-redundant mature miRNA sequences. A homology-based in silico miRNA prediction analysis was performed using this miRNA dataset as query, as previously described^40, 63, 64^. Following, a list of lncRNA transcripts and a list of coding transcripts are retrieved as target datasets for each transcriptome. These datasets were screened for relative gene targets of miRNAs, predicted from the assemblies, using psRNAtarget web-tool, with user-defined query and target options at default parameters (http://plantgrn.noble.org/psRNATarget/)^65^. lncRNAs functioning as coding-target mimics were evaluated based on the complementary pairs between miRNA-to-coding transcript targets and miRNAs-to-lncRNA targets. Cytoscape 3.3.0^66^ was used for the visualization of lncRNA-miRNA-mRNA interaction networks.

## Electronic supplementary material

Supplementary Information

Supplementary-file-1

Supplementary-file-2
