# Supplementary material for: RNA Sequencing and Co-expressed Long Non-coding RNA in Modern and Wild Wheats
Source: Sci Rep. 2017 Sep 6;7:10670. doi: 10.1038/s41598-017-11170-8 (PMC5587677; doi:10.1038/s41598-017-11170-8)
Supplement: Supplementary file 1 — Supplementary Information [file 41598_2017_11170_MOESM1_ESM.pdf]

# RNA SEQUENCING and CO-EXPRESSED LONG NON-CODING RNA IN MODERN AND WILD WHEATS

Halise Busra Cagirici[1], Burcu Alptekin[2] and Hikmet Budak[1,2]

## Supplementary Information

**Supplementary Figure 1.** *The distribution of percent change of the number of transcripts over a set of FPKM cutoffs.* Plot of percent change over 0 to 2 FPKM cutoffs **(A)**. Closer look at the graph between the cutoffs of 0 to 1 **(B)**.

**Supplementary Figure 2.** *Homology pattern between T. turgidum proteins and other plants.* **(A)** The pie chart shows the distribution of top-ten plants which showed the highest homology to *T. turgidum* proteins. **(B)** Pie chart shows the distribution of e-values for different blast hits.

**Supplementary Figure 3.** *Length distribution of lncRNAs and coding transcripts from each T. turgidum variety*. (Explanation for graph legend: Kiz-lnc: lncRNAs from variety Kiziltan, TR-lnc: lncRNAs from variety TR39477, TTD-lnc: lncRNAs from variety TTD-22, Kiz-cod: coding transcripts from variety Kiziltan, TR-cod: coding transcripts from variety TR39477, TTD-cod: coding transcripts from variety TTD-22)

**Supplementary Figure 4.** *Association between GC% content and length of lncRNAs from each T. turgidum variety.*

**Supplementary Table 1.** *TR39477 and TTD-22 specific transcripts expressed under drought stress.* **(A)** and **(B)** Drought specific transcripts of TR39477 and TTD-22 were blasted and the transcripts which do not exhibit any similarity to each other were listed in below. **(C)** KEGG maps for TR39477 specific transcripts which are expressed in response to drought stress are listed.

**Supplementary Table 2.** *miRNAs which targets the lncRNAs from variety Kiziltan* **(A)** *TR39477***(B)** *and TTD-22***(C)**.

**Supplementary Table 3.** *Number of transcripts across the three T. turgidum varities.* Number of transcripts listed either without filtering or with a filtering of >0.5 FPKM.

**Supplementary Table 4.** *QRT-PCR primers for validation of mRNA and lncRNA transcripts from Kiziltan.*

**Supplementary Table 5.** *Stem-loop RT-PCR primers for miR1436-1 and miR1436-4 from Kiziltan.*

**Supplementary File 1** *(xlsx). The annotation of transcripts from each sample.* First sheet contains the information for transcripts from variety Kiziltan. Second sheet contains the information for transcripts from variety TR39477. Third sheet contains the information for transcripts from variety TTD-22.

**Supplementary File 2** *(xlsx). miRNA-lncRNA target pairs utilized in miRNA-lncRNA-mRNA networks.* First sheet contains the information for miRNA-lncRNA pairs from variety Kiziltan. Second sheet contains the information for miRNA-lncRNA pairs from variety TR39477. Third sheet contains the information for miRNA-lncRNA pairs from variety TTD-22.

## Supplementary Figures

**Supplementary Figure 1.** *The distribution of percent change of the number of transcripts over a set of FPKM cutoffs.* Plot of percent change over 0 to 2 FPKM cutoffs **(A)**. Closer look at the graph between the cutoffs of 0 to 1 **(B)**.

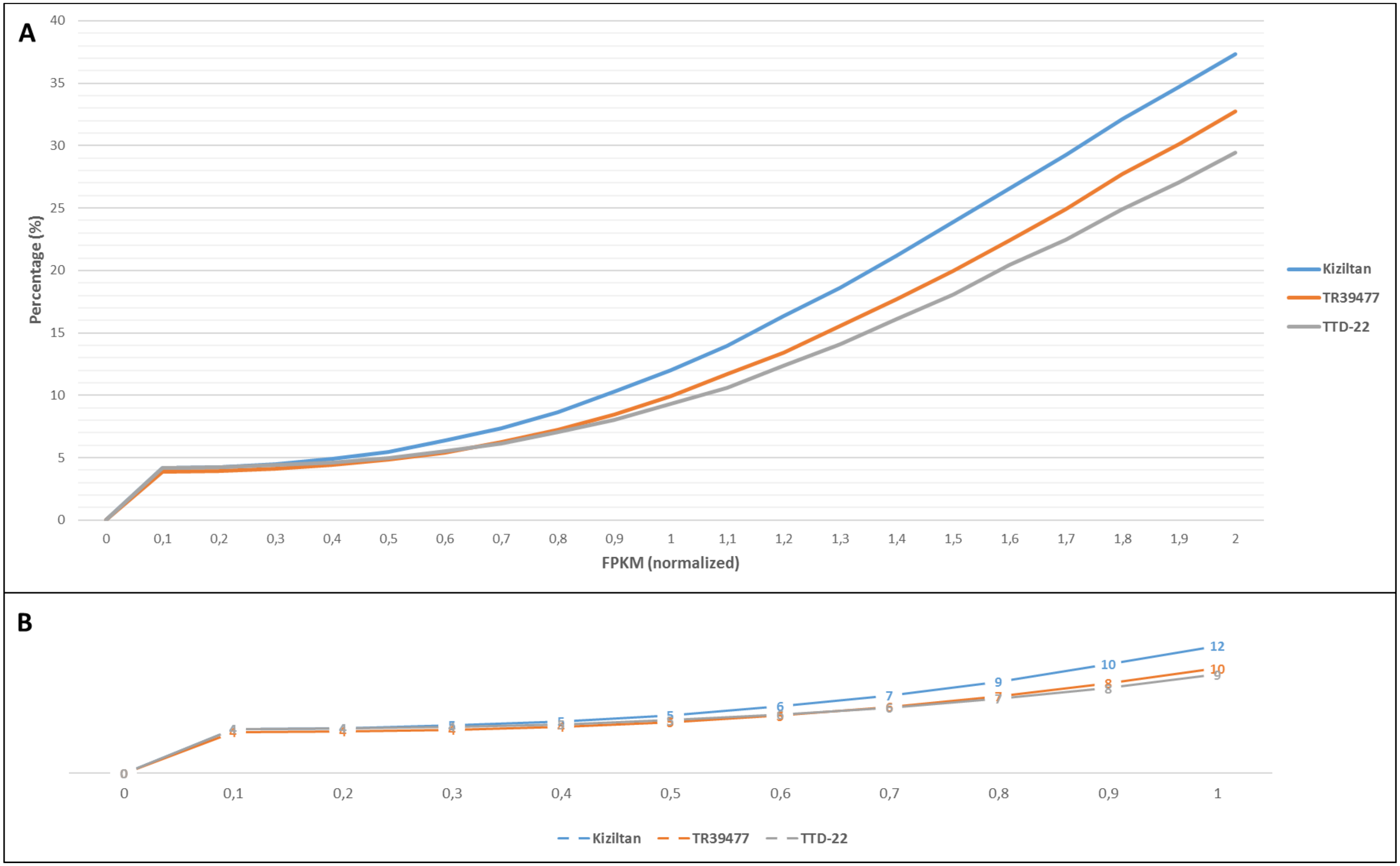

**Supplementary Figure 2.** *Homology pattern between T. turgidum proteins and other plants.* **(A)** The pie chart shows the distribution of top-ten plants which showed the highest homology to *T. turgidum* proteins. **(B)** Pie chart shows the distribution of e-values for different blast hits.

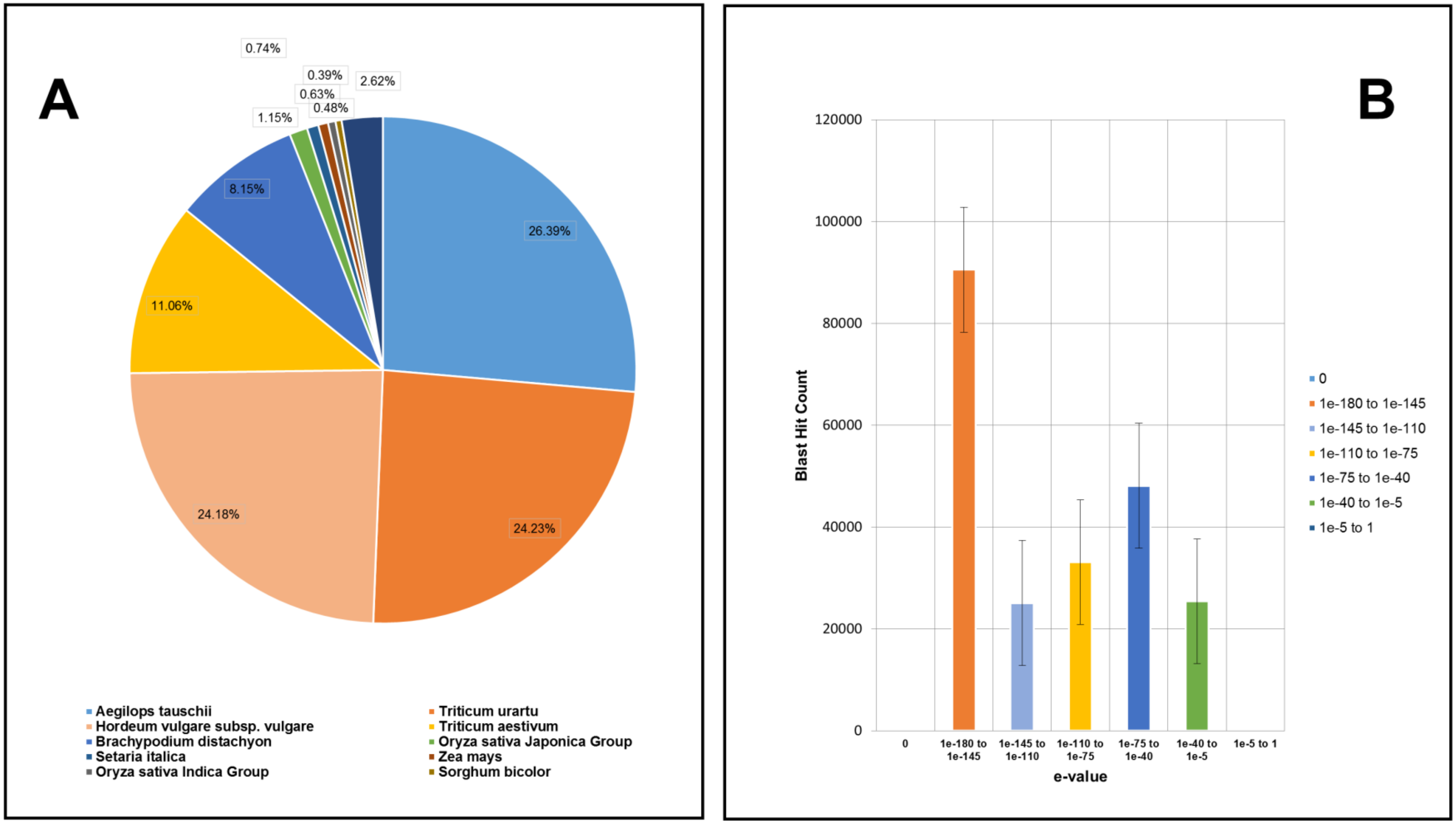

**Supplementary Figure 3.** *Length distribution of lncRNAs and coding transcripts from each T. turgidum variety*. (Explanation for graph legend: Kiz-lnc: lncRNAs from variety Kiziltan, TR-lnc: lncRNAs from variety TR39477, TTD-lnc: lncRNAs from variety TTD-22, Kiz-cod: coding transcripts from variety Kiziltan, TR-cod: coding transcripts from variety TR39477, TTD-cod: coding transcripts from variety TTD-22)

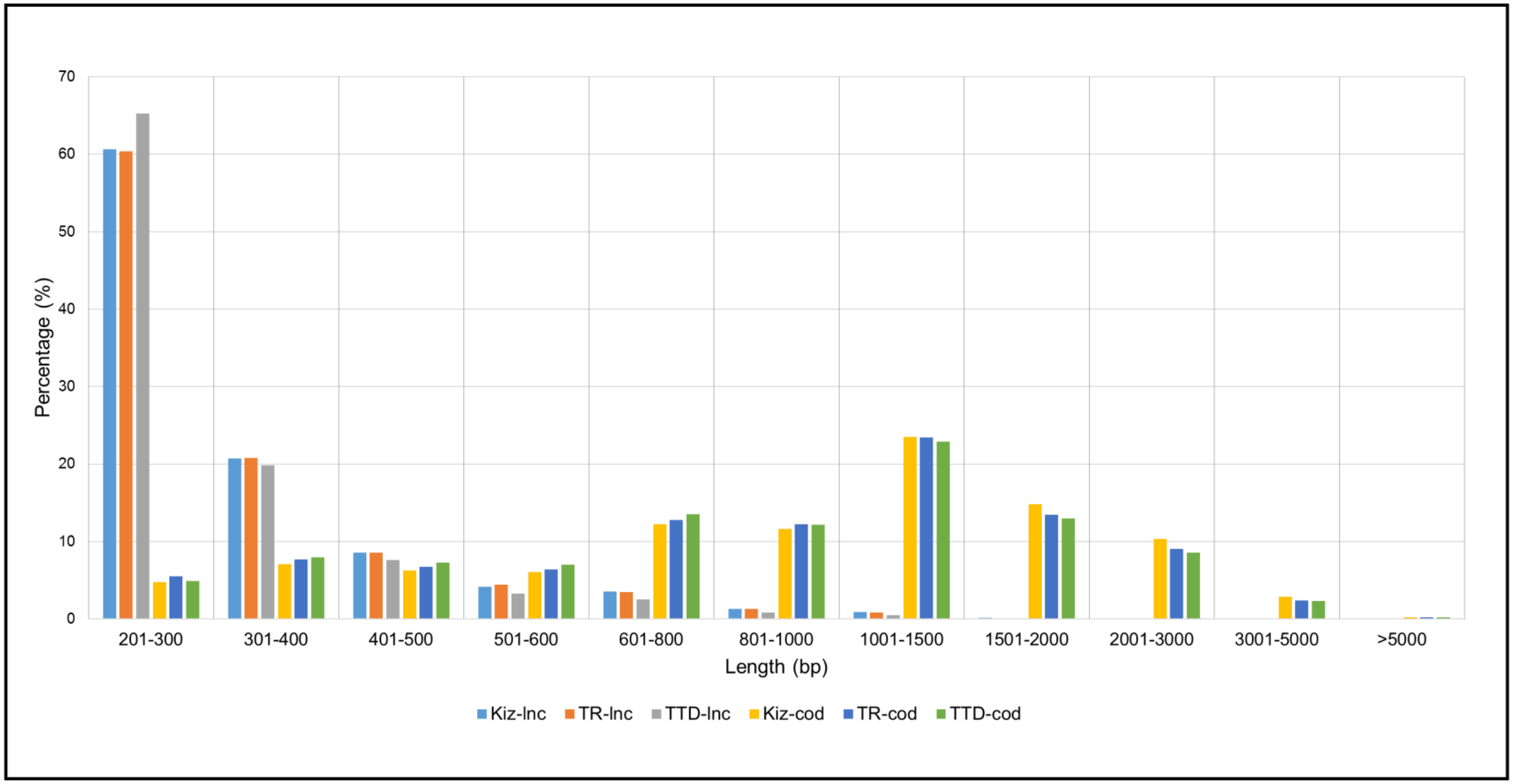

**Supplementary Figure 4.** *Association between GC% content and length of lncRNAs from each T. turgidum variety.*

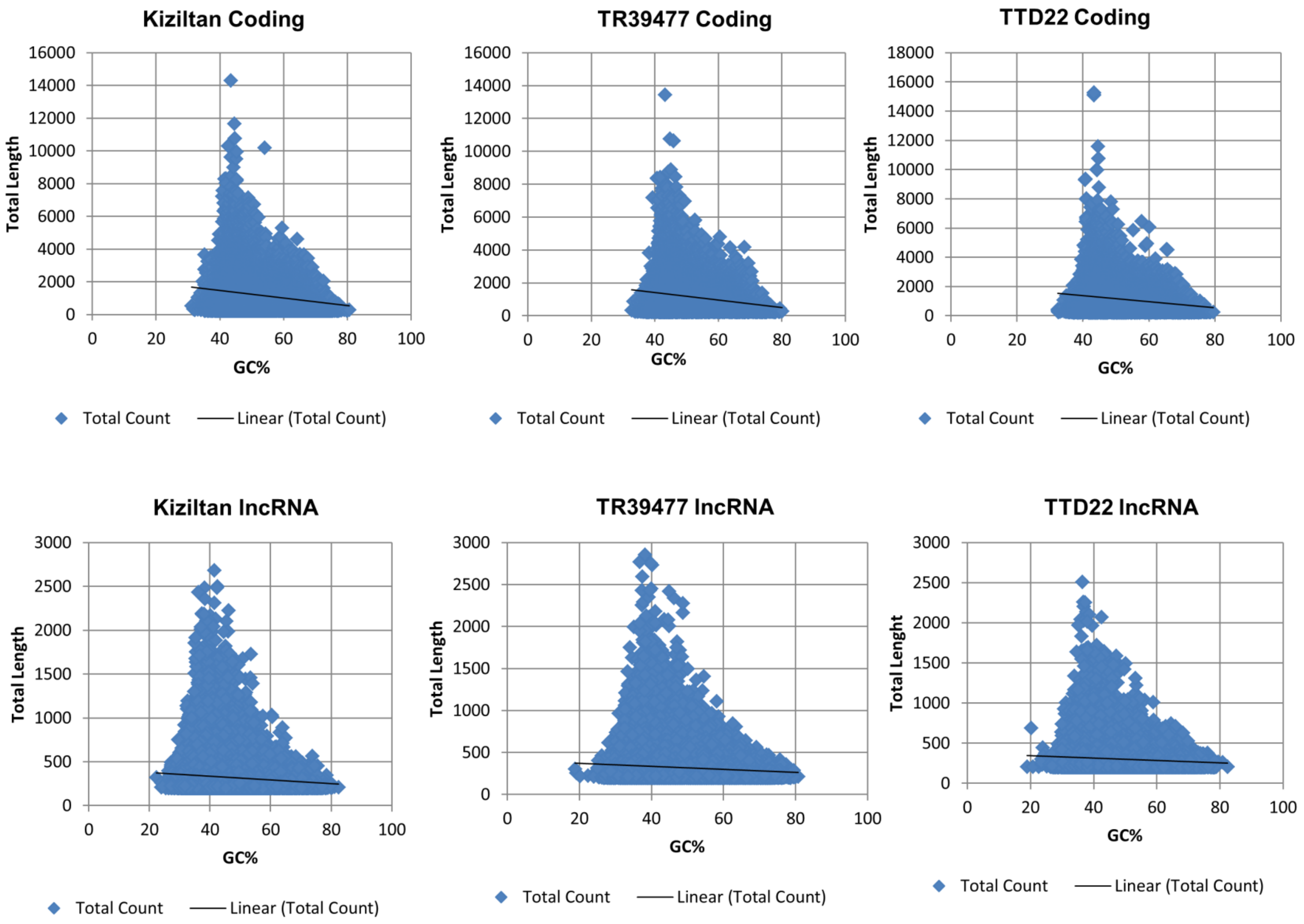

## Supplementary Tables

**Supplementary Table 1.** *TR39477 and TTD-22 specific transcripts expressed under drought stress.* **(A)** and **(B)** Drought specific transcripts of TR39477 and TTD-22 were blasted and the transcripts which do not exhibit any similarity to each other were listed in below. **(C)** KEGG maps for TR39477 specific transcripts which are expressed in response to drought stress are listed.

| | |
|---|---|
| **(A) Transcript IDs for drought specific coding transcripts of TR39477 which do not exhibit any similarity to TTD-22 transcripts** | |
| TR_DS_c100335_g1_i1,TR_DS_c100721_g1_i1,TR_DS_c10663_g1_i1,TR_DS_c111097_g1_i1,TR_DS_c112175_g1_i1,TR_DS_c115342_g1_i1,TR_DS_c118586_g1_i1,TR_DS_c119351_g1_i1,TR_DS_c121962_g1_i1,TR_DS_c122655_g1_i1,TR_DS_c124109_g1_i1,TR_DS_c133829_g1_i1,TR_DS_c139001_g1_i1,TR_DS_c13972_g1_i1,TR_DS_c18086_g1_i1,TR_DS_c23531_g1_i1,TR_DS_c32200_g1_i2,TR_DS_c5817_g1_i1,TR_DS_c59003_g1_i2,TR_DS_c68118_g1_i1,TR_DS_c7239_g1_i1,TR_DS_c76507_g1_i1,TR_DS_c77362_g1_i1,TR_DS_c77721_g1_i1,TR_DS_c79749_g1_i1,TR_DS_c81581_g1_i1,TR_DS_c83024_g1_i1,TR_DS_c83819_g1_i1,TR_DS_c84249_g1_i1,TR_DS_c86337_g1_i1,TR_DS_c87167_g1_i1,TR_DS_c87458_g1_i1,TR_DS_c87491_g1_i1,TR_DS_c93476_g1_i1,TR_DS_c94439_g1_i1,TR_DS_c9969_g1_i1 | |
| **(B) Transcript IDs for drought specific coding transcripts of TTD-22 which do not exhibit any similarity to TR39477 transcripts** | |
| TD_DS_c115797_g1_i1, TTD_DS_c53857_g1_i1, TTD_DS_c54409_g1_i4, TTD_DS_c86980_g1_i1 | |
| **(C) KEGG maps for TR39744 specific transcripts (expressed only under drought stress) and their description** | |
| map00190 | oxidative phosphorylation |
| map00480 | Glutathione metabolism |
| map00230 | Purine metabolism |
| map00600 | Sphingolipid metabolism |
| map00240 | Pyrimidine metabolism |
| map00730 | Thiamine metabolism |
| map00604 | Glycosphingolipid biosynthesis - ganglio series |
| map00531 | Glycosaminoglycan degradation |
| map00511 | Other glycan degradation |
| map00052 | Galactose metabolism |

**Supplementary Table 2.** *miRNAs which targets the lncRNAs from variety Kiziltan* **(A)** *TR39477***(B)** *and TTD-22***(C)**.

| (A) miRNAs and their lncRNAs targets from Kiziltan variety | | | | | |
|---|---|---|---|---|---|
| **miRNA ID** | **lncRNA ID** | **Target Start** | **Target End** | **Aligned Target Fragment** | **Target Inhibition Mode** |
| *miR1436* | Kiz_both_c123855_g1_i1 | 150 | 169 | CUUCUUCCAUCCCAUGAUUG | Cleavage |
| *miR1136* | Kiz_both_c38021_g1_i1 | 3 | 26 | UAGAUACAUCCAUUUCUGCGAUGA | Cleavage or Translation repression |
| *miR854* | Kiz_both_c50149_g1_i1 | 577 | 597 | CUUCUUCUUCCUCUUCUUCUU | Cleavage |
| *miR1135* | Kiz_CK_c50944_g2_i1 | 496 | 519 | UCCUUUCCAAAUUACUUGUCGUGG | Cleavage |
| *miR1439* | Kiz_CK_c50944_g2_i1 | 488 | 507 | CUACUCCCUCCUUUCCAAAU | Translation repression |
| *miR1133* | Kiz_DS_c68120_g9_i2 | 120 | 141 | UUAGGAACGGAGGGAGUAGGUC | Cleavage |
| *miR1436* | Kiz_both_c70772_g2_i1 | 132 | 151 | ACUCCCUCCGUUCC-UAAAUA | Cleavage |
| *miR1439* | Kiz_both_c70772_g2_i1 | 130 | 149 | CUACUCCCUCCGUUCCUAAA | Cleavage |
| *miR1439* | Kiz_DS_c79679_g1_i1 | 201 | 221 | [A]CUACUCCCUCCGUUCCGAAU | Cleavage |
| *miR1436* | Kiz_DS_c90557_g1_i1 | 361 | 381 | ACUCCCUCCGUUCCUUUAUGU | Cleavage |
| *miR1436* | Kiz_both_c96195_g1_i1 | 262 | 282 | ACUCCCUUUGUUCCAGAAUAA | Cleavage |
| *miR1439* | Kiz_both_c96195_g1_i1 | 259 | 279 | UUUACUCCCUUUGUUCCAGAA | Cleavage |
| *miR437* | | 304 | 324 | AACUCAAUCUUGUUUAAGUUU | Translation repression |
| **(B) miRNAs and their lncRNAs targets from TR39477 variety** | | | | | |

| miRNA ID | lncRNA ID | Target Start | Target End | Aligned Target Fragment | Target Inhibition Mode |
|---|---|---|---|---|---|
| *miR1137* | TR_both_c118135_g1_i1 | 189 | 209 | AGUGUCUCAAAUUUUGUACUA | Translation repression |
| *miR1436* | TR_both_c118135_g1_i1 | 169 | 189 | ACUACCUCCGUCCUAAAAUAA | Cleavage |
| *miR1439* | TR_both_c118135_g1_i1 | 140 | 160 | GAUACUCCCUCCGUCUUAAAA | Cleavage |
| *miR1439* | TR_both_c118135_g1_i1 | 167 | 186 | UUACUACCUCCGUCCUAAAA | Cleavage |
| *miR1436* | TR_CK_c12385_g1_i1 | 275 | 295 | ACUCCCUUUGUCUUAAAAUGA | Cleavage |
| *miR1120* | TR_DS_c34320_g1_i1 | 121 | 143 | UCCGUUUCAUAAUAUAACAGCGU | Cleavage |
| *miR1436* | TR_DS_c34320_g1_i1 | 115 | 135 | [A]CUUCAUCCGUUUCAUAAUAU | Cleavage |
| *miR1439* | TR_DS_c34320_g1_i1 | 113 | 132 | GUACUUCAUCCGUUUCAUAA | Cleavage |
| *miR1128* | TR_DS_c44260_g1_i1 | 328 | 348 | UUAGGGACGGAGGGAGUAGUU | Cleavage |
| *miR1128* | TR_both_c56781_g1_i5 | 1266 | 1285 | UUUAUAUGGAGGGAGUAUUU | Cleavage |
| *miR1133* | TR_both_c56781_g1_i1 | 1266 | 1286 | UUUAUAUGGAGGGAGUAUUUA | Cleavage |
| *miR1436* | TR_DS_c58890_g4_i2 | 448 | 467 | CUCCCUCCCUCUUUUAAUAU | Cleavage |
| *miR1139* | TR_both_c60784_g1_i5 | 461 | 480 | GUUACUAG-CUAAGUUACUCC | Cleavage |
| *miR1128* | TR_CK_c61658_g1_i1 | 4 | 24 | UUCGGAACGGAGGGAGUAGUA | Cleavage |
| *miR1436* | TR_both_c63034_g2_i23 | 24 | 44 | ACUUCCUCGGUUCCAAAAUUC | Cleavage |

| | | | | | |
|---|---|---|---|---|---|
| *miR1439* | TR_both_c63034_g2_i23 | 22 | 41 | CUACUUCCUCGGUUCCAAAA | Translation repression |
| *miR1122* | TR_DS_c63034_g2_i3 | 744 | 763 | UCUAAAUGCGGAUGUAUCUA | Cleavage |
| *miR1436* | TR_DS_c63034_g2_i3 | 709 | 729 | ACUCCCUCCGUCUCAAAAUUC | Cleavage or Translation repression |
| *miR1439* | TR_DS_c63034_g2_i3 | 706 | 726 | [A]GUACUCCCUCCGUCUCAAAA | Cleavage |
| *miR1118* | TR_DS_c63271_g2_i1 | 831 | 853 | UCCCUCCAUUCCAAAAUAUAGCG | Cleavage |
| *miR1436* | TR_DS_c63271_g2_i1 | 829 | 849 | [A]CUCCCUCCAUUCCAAAAUAU | Cleavage |
| *miR1439* | TR_DS_c63271_g2_i1 | 826 | 846 | [C]UUACUCCCUCCAUUCCAAAA | Cleavage or Translation repression |
| *miR1136* | TR_DS_c63631_g1_i7 | 662 | 681 | AACAUUCAUAUGUGUGACAU | Cleavage |
| *miR1436* | TR_DS_c65082_g1_i1 | 244 | 264 | ACUCCCUCCGUUCCUUUAUAU | Cleavage |
| *miR1439* | TR_CK_c67474_g1_i1 | 401 | 421 | ACUGCUCCCUCCGUUUCUAAA | Cleavage |
| *miR1130* | TR_both_c94590_g1_i1 | 210 | 232 | [AU]UCUUAUAUUAUGGGACGGAGG | Cleavage or Translation repression |
| *miR1436* | TR_both_c94590_g1_i1 | 188 | 207 | ACUCCUUCUGUCCC-UAAUGC | Cleavage |
| **(C) miRNAs and their lncRNAs targets from TTD-22 variety** | | | | | |
| **miRNA ID** | **lncRNA ID** | **Target Start** | **Target End** | **Aligned Target Fragment** | **Target Inhibition Mode** |
| *miR1436* | TTD_DS_c58970_g2_i3 | 225 | 245 | [A]CUCCCUCCGUUCCAAAAUAG | Cleavage |
| *miR1439* | TTD_DS_c58970_g2_i3 | 223 | 242 | [G]UACUCCCUCCGUUCCAAAA | Cleavage |
| *miR1128* | TTD_DS_c60722_g1 | 4 | 24 | UUUGGGACGGAGGGAGUACU | Cleavage |

| | | | | | |
|---|---|---|---|---|---|
| | _i1 | | | A | |
| *miR1120* | TTD_CK_c62154_g1_i2 | 682 | 705 | CUCCGUCCCAUAAUAUAACAGCGU | Cleavage |
| *miR1436* | TTD_CK_c62154_g1_i2 | 677 | 697 | [A]UUCCCUCCGUCCCAUAAUAU | Cleavage or Translation repression |
| *miR1436* | TTD_DS_c64640_g2_i1 | 2490 | 2510 | ACUCCCUCCGUCCCAAAAUUC | Cleavage |
| *miR1439* | TTD_DS_c64640_g2_i1 | 2487 | 2507 | [A]CUACUCCCUCCGUCCCAAAA | Cleavage |

**Supplementary Table 3.** *Number of transcripts across the three T. turgidum varities.* Number of transcripts listed either without filtering or with a filtering of >0.5 FPKM.

| | The number of | Kiziltan | TR39477 | TTD-22 |
|---|---|---|---|---|
| Without filtering | All transcripts | 243670 | 211709 | 203230 |
| | Coding transcripts | 84288 | 75996 | 78456 |
| | lncRNA transcripts | 63773 | 61823 | 43932 |
| Actively-expressed | All transcripts | 230359 | 201499 | 193087 |
| | Coding transcripts | 81168 | 73465 | 75861 |
| | lncRNA transcripts | 59110 | 57944 | 40858 |

**Supplementary Table 4.** *QRT-PCR primers for validation of mRNA and lncRNA transcripts from Kiziltan.*

| lncRNA ID | lncRNA Length | Sequence | Primer Start Site in Target | Primer End Site in Target | Length | GC % | Amplicon Length |
|---|---|---|---|---|---|---|---|
| lncRNA_c118446_g1_i1-Forward | 341 | TCTCTGGCCTAAGCAACTTTAC | 100 | 122 | 22 | 45.5 | 127 |
| lncRNA_c118446_g1_i1-Reverse | 341 | GCTTTCCCAAAGCCCTGATA | 207 | 227 | 20 | 50 | 127 |
| lncRNA_c47700_g1_i1-Forward | 472 | GGAACAGCGACAGTACAGTAAG | 188 | 210 | 22 | 50 | 139 |
| lncRNA_c47700_g1_i1-Reverse | 472 | TGTGTGACTGTGAGAGAGAGATA | 304 | 327 | 23 | 43.5 | 139 |
| mRNA_c17408_g1_i1-Forward | 879 | CTCAGACCTTCGATCAAAGACG | 110 | 132 | 22 | 50 | 97 |
| mRNA_c17408_g1_i1-Reverse | 879 | TCCATGTACGTCCACCTAGAG | 186 | 207 | 21 | 52.4 | 97 |
| mRNA_c55246_g1_i1-Forward | 1734 | CGACGTGTAAGCATCAGAGAA | 154 | 175 | 21 | 47.6 | 105 |
| mRNA_c55246_g1_i1-Reverse | 1734 | AGCCTATGCACTTCCCTAAATC | 237 | 259 | 22 | 45.5 | 105 |
| lncRNA_c70772_g2_i1-Forward | 208 | GCCTCCCTGTGCAAACATA | 81 | 100 | 19 | 52.6 | 102 |
| lncRNA_c70772_g2_i1-Reverse | 208 | AGTCACTTGTTGAAATGCCTAGA | 160 | 182 | 23 | 39.1 | 102 |
| mRNA_c69036_g1_i1-Forward | 756 | ATTGGCGCTGACGAGAAA | 251 | 269 | 18 | 50 | 98 |
| mRNA_c69036_g1_i1-Reverse | 756 | CTACCTGCACAGTCCATCTTC | 328 | 349 | 21 | 52.6 | 98 |
| lncRNA_c90557_g1_i1-Forward | 385 | CTGCTCTGCTCTGTACTGTATC | 104 | 126 | 22 | 62 | 88 |
| lncRNA_c90557_g1_i1-Reverse | 385 | GATCTCCTCTACAGCCACAAG | 171 | 192 | 21 | 61 | 88 |
| mRNA_ c9653_g1_i2-Forward | 2483 | ATCCTCTCCTTCCTGGTCAA | 872 | 892 | 20 | 50 | 92 |
| mRNA_ c9653_g1_i2-Reverse | 2483 | CCTCGACTGCGTGAAATAGAA | 943 | 964 | 21 | 47.6 | 92 |

**Supplementary Table 5.** *Stem-loop RT-PCR primers for miR1436-1 and miR1436-4 from Kiziltan.*

| **Primer Name** | **Sequence** |
|---|---|
| Universal Reverse Primer for qRT-PCR | GTGCAGGGTCCGAGGT |
| miR1439-3_Stemloop | GTTGGCTCTGGTGCAGGGTCCGAGGTATTCGCACCAGAGCCAACAATACT |
| miR1436-1_Stemloop | GTTGGCTCTGGTGCAGGGTCCGAGGTATTCGCACCAGAGCCAACACTCCC |
| miR1436-4_Stemloop | GTTGGCTCTGGTGCAGGGTCCGAGGTATTCGCACCAGAGCCAACACTCCC |
| miR854_Stemloop | GTTGGCTCTGGTGCAGGGTCCGAGGTATTCGCACCAGAGCCAACCTCCTC |
| miR1133-1_Stemloop | GTTGGCTCTGGTGCAGGGTCCGAGGTATTCGCACCAGAGCCAACTTTGGG |
